# Bypassing the requirement for aminoacyl-tRNA by a cyclodipeptide synthase enzyme†

**DOI:** 10.1039/d0cb00142b

**Published:** 2021-01-15

**Authors:** Christopher J. Harding, Emmajay Sutherland, Jane G. Hanna, Douglas R. Houston, Clarissa M. Czekster

**Affiliations:** School of Biology, Biomedical Sciences Research Complex, University of St Andrews, St Andrews Fife KY16 9ST UK cmc27@st-andrews.ac.uk; Arab Academy for Science, Technology, and Maritime Transport (AASTMT) Cairo Campus Egypt; Institute of Quantitative Biology, Biochemistry and Biotechnology, University of Edinburgh Waddington 1 Building, King's Buildings Edinburgh EH9 3BF UK

## Abstract

Cyclodipeptide synthases (CDPSs) produce a variety of cyclic dipeptide products by utilising two aminoacylated tRNA substrates. We sought to investigate the minimal requirements for substrate usage in this class of enzymes as the relationship between CDPSs and their substrates remains elusive. Here, we investigated the *Bacillus thermoamylovorans* enzyme, BtCDPS, which synthesises cyclo(l-Leu–l-Leu). We systematically tested where specificity arises and, in the process, uncovered small molecules (activated amino esters) that will suffice as substrates, although catalytically poor. We solved the structure of BtCDPS to 1.7 Å and combining crystallography, enzymatic assays and substrate docking experiments propose a model for how the minimal substrates interact with the enzyme. This work is the first report of a CDPS enzyme utilizing a molecule other than aa-tRNA as a substrate; providing insights into substrate requirements and setting the stage for the design of improved simpler substrates.

## Abstract 2

Cyclodipeptide synthases recognize a minimalistic substrate to produce cyclic dipeptides in a tRNA-independent manner.

## Graphical abstract

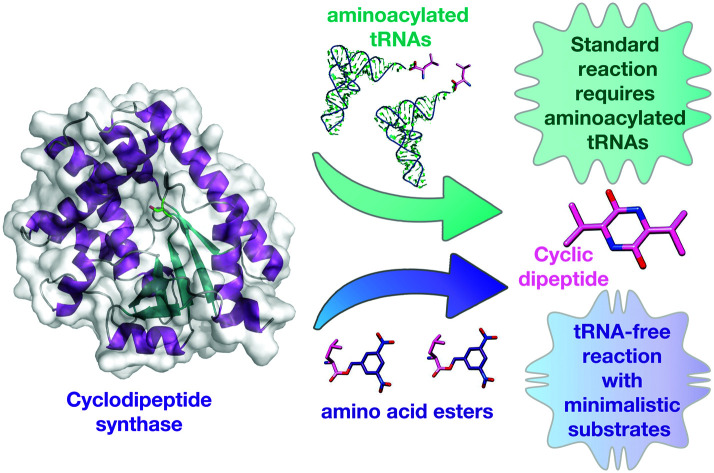

## Introduction

Cyclodipeptide synthases (CDPS) are a family of tRNA dependant enzymes that synthesise cyclic dipeptides using two aminoacylated tRNA (aa-tRNA) as substrates.^1–3^ Cyclic dipeptides are members of a large variety of 2,5-diketopiperazine-containing natural products, which can be synthesized *via* non-ribosomal peptide synthetases or using CDPS enzymes.^4,5^ They are produced by organisms in all domains of life, and thought to interfere with interspecies and interkingdom relationships, albeit by unknown mechanisms.^6,7^ Importantly, cyclic dipeptides possess a privileged chemical structure, conferring high protease and temperature stability, easy absorption by the gut and the ability to cross the blood brain barrier, all invaluable therapeutic traits.^4^ Important biological activities have been described for several cyclic dipeptides, including antifungal,^9^ antibacterial,^10^ antitumor,^11^ antiviral,^12^ immunosuppressant^13^ and quorum sensing properties.^14^ Once produced, cyclic dipeptides can be highly modified by tailoring enzymes to produce more complex natural products such as mycocyclosin^15^ and bicyclomycin,^16,17^ amongst others.^5^

**Fig. 1:**
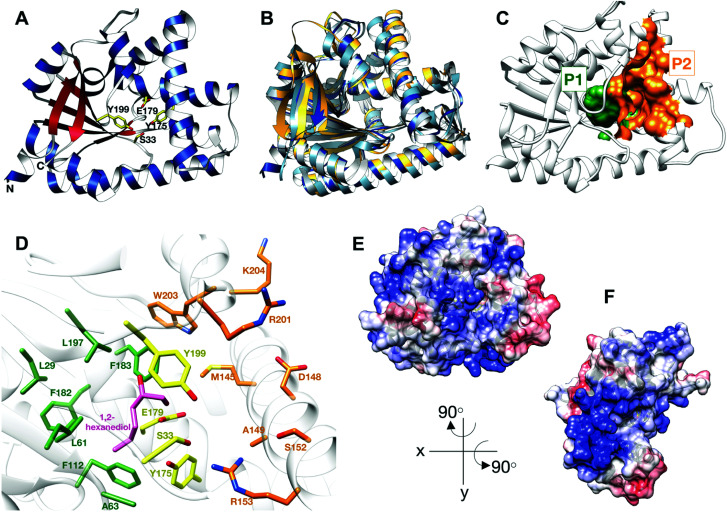
Structure of BtCDPS (A) The overall structure BtCDPS in ribbon form, alpha-helices (blue), beta-strands (red) and active site residues (yellow). BtCDPS has an alpha-beta Rossmann fold. (B) Comparison to CDPS homologue structures, which superpose well by secondary structure alignment. BtCDPS – dark blue, Rv2275 (2X9Q) – yellow, AlbC (4Q24) – light blue, YvmC-Blic (3OQI) – grey. (C) Substrate binding pockets P1 and P2. (D) The residues of the substrate binding pockets of BtCDPS are coloured green for P1, orange for P2 and the catalytic residues are coloured yellow. A 1,2-hexanediol bound to the substrate binding pocket of BtCDPS is shown in stick representation (pink). The deeper P1 pocket is lined with hydrophobic residues, whereas the shallower P2 pocket has more variability in residues. Arg153 faces into the active site and make an unconventional H-bond with the catalytic Ser33 residue. (E and F) The Electrostatic surface potential of BtCDPS mapped onto the solvent-accessible surface at a contour of ±10 kcal mol^−1^ e^−1^ using Chimera.^8^ Positive charge is in blue and negative charge is in red. Two patches of positive charges exist beneath the substrate binding pocket.

CDPS were first identified in 2002 and have since received much attention.^18^ All CDPS structurally characterised to date share a single αβ domain containing a Rossmann fold.^19–22^ They are structurally related to the catalytic domain of class-Ic aa-tRNA synthetases, such as tyrosyl- and tryptophanyl-tRNA synthetases, suggesting an evolutionary link.^21^ However, CDPS differ significantly from class-Ic aa-tRNA synthetases by lacking an ATP-binding motif and function as active monomers, rather than homodimers. The structures of CDPS enzymes have two deep solvent accessible pockets (P1 and P2), neighbouring the catalytic site, believed to accommodate the aminoacyl moiety of aa-tRNA substrates (Fig. 1C and D).^23^

The catalytic mechanism for these enzymes has been well documented. CDPS use two aa-tRNA substrates in a ping-pong mechanism, which forms two successive acyl–enzyme intermediates.^19–21,24^ The aminoacyl moiety of tRNA is transferred onto the conserved active site serine residue (S33 in BtCDPS, Fig. 1D), forming an aminoacyl-enzyme intermediate.^19–21,25^ This intermediate then reacts with a second aa-tRNA to form a dipeptidyl-enzyme intermediate, which undergoes intramolecular cyclisation aided by a conserved tyrosine residue to form a cyclic dipeptide.^23,26^

CDPS are fairly promiscuous enzymes that predominantly synthesise one main cyclodipeptide product and several other cyclodipeptides to a lesser extent.^2^ Typically, CDPS favour a single aminoacyl moiety, which is accommodated in the P1 binding pocket and have less specificity for the second substrate, which binds less stringently into the shallower P2 pocket. For instance, the major product of AlbC from *Streptomyces noursei* is cFL, although significant amounts of cFX (where X represents another amino acid) are also produced. Additionally, Rv2275 from *Mycobacterium tuberculosis* predominantly synthesises cYY, yet other cYX products are possible.^1,2^ The residues lining the P1 and P2 pockets confer specificity for the substrates, which has allowed early prediction of the cyclodipeptide produced with limited success.^1,2^ Binding of the tRNA moiety is thought to be less important to substrate selection, where patches of charged residues on the surface of CDPS are predicted to facilitate binding.^20,21^ However, preference towards different tRNA^Leu^ isoacceptors by AlbC led to speculation that specific base pairing on the acceptor stem may also be essential for recognition.^24^ A recent study provided important insights into the interaction between protein and aa-tRNA.^27^ However, the mechanism by which CDPS select and recognise their specific set of substrates from the pools of aa-tRNA remains ambiguous as a high resolution enzyme-substrate complex is still lacking.

Here, we investigated a CDPS from *Bacillus thermoamylovorans* (BtCDPS), a cLL-synthesising enzyme that utilises Leu-tRNA^Leu^ as a substrate, and is predicted to be part of the biosynthetic machinery involved in pulcherrimin biosynthesis.^20^ We set out to explore the minimal requirements for substrate usage by systematically testing whether the aminoacyl moiety, the tRNA moiety or both are fundamental for catalysis. In the process we uncovered that a small molecule – an activated amino ester – will suffice as a substrate. This work is the first report of a CDPS enzyme utilizing a small molecule as a substrate. Our work provides crucial insights into substrate requirements and lays the foundation for the design of simpler substrates as well as CDPS enzyme variants with improved catalytic properties using minimal substrates.

## Results and discussion

### Structure of wild type BtCDPS

Thermostability is an attractive property for enzymes to possess. Thermophilic enzymes offer simpler strategies for protein isolation and regeneration,^28^ and have been shown to possess higher tolerance to harsher reaction conditions, for example organic solvents.^29^ As no thermostable CDPS has been characterized, and only two have been described to date, we focused on the CDPS from *Bacillus thermoamylovorans* (BtCDPS). BtCDPS shares 50% sequence identity to yvmC-BLIC from *Bacillus licheniformis* (another cLL synthesising CDPS),^20^ and has a melting temperature of 66 °C (Fig. S8, ESI†). The crystal structure of full-length (residues 1–232) and S33A mutant enzymes was solved at a resolution of 1.69 Å and 1.78 Å, respectively (Fig. 1 and Fig. S13, ESI†). BtCDPS has a single domain with an αβ fold similar to other CDPS.^19–22^ The structure of CDPS is highly similar to yvmC-BLIC (RMSD 0.88 Å, over 212 residues), AlbC (RMSD 2.25, over 212 residues) and Rv2275 (RMSD 2.00 Å, over 199 residues) (Fig. 1B). A molecule of 1,2-hexanediol co-crystallised in the P1 binding pocket adjacent to the catalytic residues (Fig. 1D). Binding is facilitated by a hydrogen bond contact with the side chain carboxyl group of the catalytic Glu179 residue and other non-bonding contacts with P1 pocket residues. The aliphatic chain of 1,2-hexanediol molecule is projected deep into the P1 pocket, which is lined with mainly hydrophobic residues (L29, G31, L61, A63, F112, Phe182, F183, L197), ideal to accommodate a leucine substrate. However, the P2 pocket is far shallower and more solvent exposed (Fig. 1C). The P2 pocket is lined with a mixture of residues (R201, W203, K204, D148, S152, A149, R153), which is in keeping with the previously reported sequence homology that suggests the P2 pocket has conserved Met, Trp and Ala residues.^2^ Interestingly, superimposition of yvmC-BLIC on to the structure of BtCDPS, reveals Arg153 may have a potential role in substrate binding. The side chain of R153 is projected into the active site and makes a strong hydrogen-bond (distance = 2.6 Å) with the hydroxyl side chain of the catalytic serine residue, which is unknown for this class of enzymes (Fig. 1D). Moreover, the positioning of R153 makes it an ideal candidate to interact with the 5′-phosphate group of the aa-AMP moiety of tRNA substrate. Intriguingly, the CHES molecule in the yvmC-BLIC complex structure (3OQI) is positioned such that its sulphate group is in close proximity to R153, therefore a strong bidentate ionic bond would occur (Fig. S12, ESI†). Further examination of the crystal structure of BtCDPS suggests that there are two patches of basic residues on its surface (Fig. 1E and F), which could be involved in binding to the tRNA body. Similar basic patches have also been documented in other CDPS, such as AlbC, where mutations of the basic residues led to decreased production of cyclodipeptides.^24^

### Substrate tolerance for tRNA isoacceptor and amino acid moiety

**Fig. 2:**
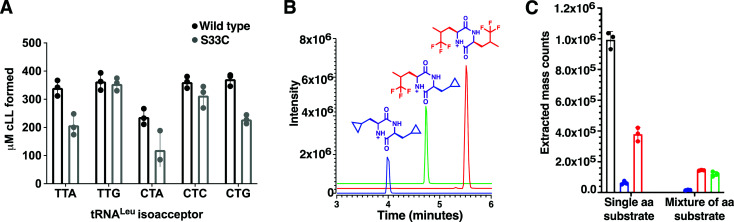
Substrate selection (A) Amount of cLL detected using an endpoint assay, monitoring BtCDPS preference for different tRNA^Leu^ isoacceptors. There is no significant difference between tRNA^Leu^ isoacceptors. The BtCDPS_S33C_ mutant was also similarly as active as wild type under assay conditions. (B) Products produced by BtCDPS. Chromatogram of the extracted mass for each product. Graph shows the integrated peak area for each product. (C) Results obtained when the assay was performed in the presence of either a single amino acid (leucine, cyclo-penta-alanine or tri-fluoroleucine) or a mixture (cyclo-penta-alanine and trifluoroleucine). Leucine was used as a positive control in this experiment.

Using a coupled assay with Leucyl-tRNA synthetase to regenerate the Leu-tRNA^Leu^ substrates, a set of 5 leu-tRNA isoacceptors from *B. thermoamylovorans* (TTA, TTG, CTA, CTC, CTG) were all tested individually and produced similar quantities of cLL product in the endpoint assay, suggesting BtCDPS does not have a strong preference for the tRNA moiety (Fig. 2A). TTA, TTG, CTC and CTG isoacceptors yielded similar amounts of cLL products, whereas CTA produced slightly less than the others. This result is in partial agreement with data collected for AlbC (cFL synthesising), which demonstrated AlbC utilised different tRNA isoacceptors with different preferences, but in that case some isoacceptors were not tolerated. The G^1^–C^72^ pair on the acceptor stem was proposed to be an essential element for substrate recognition, due to the reduced activity with CTC and TTA isoacceptors. Subsequent work has indicated a more complex picture where different elements could be involved.^1^ A reduced form of aa-tRNA (a flexizyme aminoacylated minitRNA including the acceptor stem) was shown to be sufficient for cyclodipeptide synthase activity, suggesting the tRNA moiety can be altered significantly and still accepted as a substrate.^30^ Additionally, only small differences were observed for the activity of WT and a S33C mutant, while a larger impairment was observed when a similar mutation was performed in AlbC.^21^

By using mutant forms of tRNA synthetases (LeuRS-D345A^31^ and IleRS-D342A^32^) which lacked editing activity in our coupled assay, we generated tRNA^Leu^ and tRNA^Ile^ substrates misaminoacylated with either leucine, isoleucine, methionine and valine. When using misaminoacylated substrates we detected cLL, cII and cMM products, while cVV was not detected, suggesting BtCDPS can accommodate and use other similar amino acids in the active site to synthesise cyclic di-peptide products (Fig. S10, ESI†). In addition, this result also demonstrates BtCDPS is capable of accepting other tRNA bodies (*i.e.* Leu-tRNA^Ile^) as long as the loaded amino acid is also tolerated.

Leucyl-tRNA synthetase has an intrinsic promiscuity for particular unnatural leucine analogues,^33^ which we exploited to investigate the scope of BtCDPS’ specificity for the amino acid moiety of the tRNA. We swapped l-leucine for either cyclo-penta-alanine, trifluoroleucine or both cyclo-penta-alanine and trifluoroleucine in the cLL synthesising assay and used LC-MS to detect product formation. BtCDPS utilised the unnatural amino acids tested to form cyclic dipeptide products (Fig. 2B and C and Fig. S9, ESI†), demonstrating that a degree of promiscuity of the amino acid moiety is allowed. This is in similar agreement to other research that also reported other CDPS can utilise non-canonical amino acids.^34^

### Minimalistic substrate development

Puromycin is a common aminoacyl-tRNA mimic, accepted by the ribosome and other enzymes that bind aminoacyl-tRNAs.^35,36^ We therefore synthesized leucine-puromycin (Leu-PANS), in which the *O*-methyl-tyrosine amide in puromycin was replaced by a leucine, to test whether this was the case for BtCDPS. We hypothesised Leu-PANS would interact with the substrate binding pocket. However, ITC experiments (at concentrations as high as 2 mM) showed no binding occurred (Fig. S19, ESI†). In addition, we tested Leu-PANS as an inhibitor in the aminoacylated tRNA assay, but did not observe any inhibition of BtCDPS activity (concentrations up to 2 mM) (Fig. S21, ESI†), supporting our ITC experiments. We predict the ester bond linking the amino acid to the nucleotide is playing an essential role in substrate selection in CDPS enzymes.

Combining the fact that BtCDPS does not bind Leu-PANS, but accepts all Leu-tRNA^Leu^ isoacceptors and leucine analogues as substrates, we proposed the minimal substrate requirements for CDPS enzymes is an activated amino ester with a moderately stringent selection/rejection of the amino acid side chain, while the tRNA body plays a role in efficient substrate positioning. To test this hypothesis, we examined amino esters of leucine containing umbelipherone, *p*-nitrophenyl and dinitrobenzyl as leaving groups (Fig. S18, ESI†) (Leu-umb, Leu-PNP, Leu-DBE, Val-DBE and Pro-DBE, Ile-DBE and Met-DBE) as substrate for BtCDPS.

**Fig. 3:**
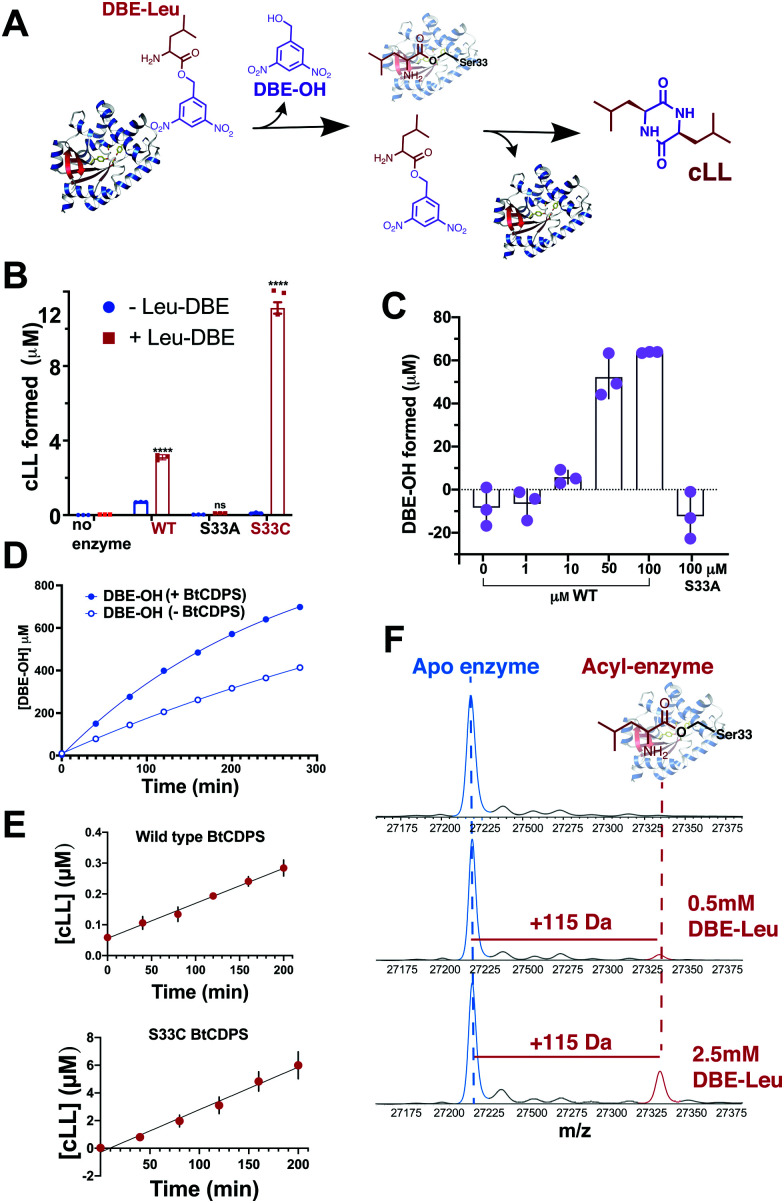
Small molecule, Leu-DBE, is a substrate of BtCDPS (A) Reaction scheme when utilising Leu-DBE as a substrate. (B) Leu-DBE assay depicting the levels of cLL (*m*/*z* = 227.175) detected by LC-HRMS. B and C are reactions performed overnight in the presence (+) or absence (−) of Leu-DBE, using wild type (WT) or active site mutants S33A and S33C. **** depicts significant differences between + and − experiments. (C) Integrated DBE-OH peaks show production dependant on concentration of enzyme. (D) Time course for DBE-OH formation in the presence (half life, *t*_1/2_ = 182 min) and absence (*t*_1/2_ = 431 min) of BtCDPS. Solid line is a fit to an exponential equation to obtain *t*_1/2_. (E) Time course for cLL formation catalysed by wild type and S33C BtCDPS. (F) Intact mass spectrometry of wild type BtCDPS shows the accumulation of a leucyl-enzyme intermediate (mass increase of +115) with increasing concentration of Leu-DBE substrate in assay. Accumulation is not observed when using S33A and S33C (Fig. S16). *n* = 3 independent experimental replicates for Leu-DBE assays.

Our results show that a cyclic dipeptide (cLL) is generated by CDPS when Leu-DBE was used as a substrate (Fig. 3B), albeit with low efficiency, while other leaving groups (coupled to leucine) that were tested were not substrates. Furthermore, in line with our experiments employing misaminoacylated tRNAs, Ile-DBE and Met-DBE were also substrates for BtCDPS (Fig. S11, ESI†). Addition of a dinitrobenzyl ester group to amino acids has been extensively used as a tool to activate amino acids and aminoacylate tRNAs, in a reaction catalysed by ribozymes of the Flexizyme family.^37,38^ Both BtCDPS WT and S33C mutant (potentially a better nucleophile, facilitating cLL production in comparison to WT due to increased reactivity of its thioester enzyme intermediate) showed a significant increase in the amount of cLL that was detected by LC-MS, whereas the S33A mutant was inactive. To identify the cause of low reaction yield (78 μM cLL product formed per μM WT BtCDPS with a Leu-tRNA^Leu^ substrate, compared to 0.8 or 2.4 μM cLL product formed per μM WT or S33C variants, respectively), and to confirm that Leu-DBE consumption was due to BtCDPS, we quantified the amount of hydrolysed product (DBE-OH) over time and in the presence and absence of increasing concentrations of enzyme. The amount of DBE-OH formed was directly dependant on increasing enzyme concentration (Fig. 3C). Moreover, DBE-OH formation and Leu-DBE hydrolysis occurred at a faster rate when BtCDPS was present (Fig. 3D and Fig. S20, respectively, ESI†). We also observed time dependent cLL production with both BtCDPS WT and S33C mutant (Fig. 3E).

### Docking studies

**Fig. 4:**
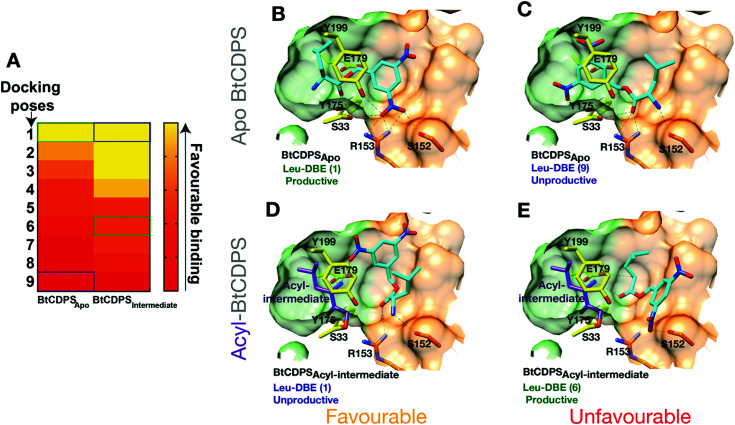
Docking of Leu-DBE to BtCDPS. Docking experiments reveal Leu-DBE binds to BtCDPS with more favourable catalytically productive poses in the first catalytic cycle compared to the second catalytic cycle. (A) A heat map illustrating relative Δ*G* values (Pose 1 most favourable – pose 10 least favourable) for docking of Leu-DBE to BtCDPS_Apo_ and BtCDPS_Intermediate_. BtCDPS_Intermediate_ represents an acyl-intermediate enzyme state created by superimposing PDB: 4Q24 and trimming down the ligand atoms to convert to leucine, before merging. (B) In the first catalytic cycle Leu-DBE (cyan) docks favourably to BtCDPS_apo_ in catalytically productive poses, represented here by pose 1. The leucine moiety places into the P1 pocket (green carbon atoms), with the amide bond place between the catalytic residues, E179, S33 and Y175 (yellow carbon atoms). The nitro groups from DBE-Leu interact with S33, Y199, R153 and S152. (C) Representation of the less favourable docking pose 9. The DBE moiety occupies the P1 pocket, thus the leucine moiety is in a catalytically unproductive pose. (D) Pose 1, represents the most favourable docking pose of Leu-DBE (cyan) to BtCDPS_Intermediate_, which is a model of a leucyl-S33 intermediate (purple carbon atoms) occupying the P1 pocket. The Leu-DBE molecule in pose 1 binds in a catalytically unproductive manner. (E) The less favourable, pose 6, represents a catalytically productive binding pose of Leu-DBE to BtCDPS_Intermediate_. The leucine moiety sits in the P2 pocket (residues with orange carbon atoms) and the amide bond is within hydrogen bonding distance of the catalytic residues.

We hypothesised that BtCDPS could use DBE-Leu to catalyse the first half reaction generating the acyl enzyme intermediate more efficiently than the second step, leading to a large proportion of enzyme becoming trapped as an acyl form. This could be due to improper substrate positioning to binding site P2, which would eventually lead to acyl–enzyme hydrolysis and a futile cycle. To test this hypothesis, we performed docking experiments using PSOVina2 (Fig. 4). Docking of a leu-DBE molecule was performed on the crystallographic structure of BtCDPS (BtCDPS_apo_) and a model of an acyl-intermediate (BtCDPS_intermediate_), which was created by superimposing PDB: 4Q24 (possesses acyl-intermediate substrate mimic (*N*-carbobenzyloxy-l-Phe-chloromethyl ketone), trimming down the ligand atoms to convert to leucine and merging with BtCDPS. Docking of Leu-DBE to BtCDPS_apo_ (representing the first catalytic cycle), mainly produced catalytically productive binding poses within the top ranked Δ*G* values (Fig. 4B). These productive poses successfully place the leucine moiety into the P1 pocket with H-bonding contacts to key catalytic residues (E179, S33 and Y175). The DBE moiety sits in the P2 pocket and the nitro-groups H-bonds with S33, Y199, S152 and interestingly to R153, which we speculated would interact with the AMP 5′-phosphate group of aa-tRNA substrates. To validate the docking experiment and test the importance of R153 to reactivity and active site architecture, we produced the mutant R153A (BtCDPS_R153A_). Structurally BtCDPS_R153A_ is extremely similar to the WT BtCDPS and BtCDPS_S33C_. However, activity with both Leu-tRNA^Leu^ and Leu-DBE is completely lost, supporting our observation that this residue likely has an important role during catalysis. Besides the loss of enzyme-substrate interactions, the structure of BtCDPS_R153A_ highlights R153 importance in correctly positioning the active site S33 sidechain (Fig. S13, ESI†). The conformation of S33 sidechain is rotated by ∼85°, which orientates it to an inward facing position, closer to the sidechain of Y199 (another catalytic residue) to form an alternative H-bond in the structure of BtCDPS_R153A_ when compared to WT BtCDPS (2.8 Å *vs.* 3.5 Å). Additionally, helix-7 is displaced (<2 Å) in the BtCDPS_R153A_, due to the absence of R153.

The most productive Leu-DBE docking pose is highly similar to the proposed aa-tRNA docking pose present in the low resolution CDPS:aa-tRNA complex, where the terminal A76 adenosine occupies the P2 pocket.^27^ Docking of Leu-DBE to the BtCDPS_intermediate_, which represents the second catalytic binding event, suggests catalytically productive poses are less favourable than non-productive poses. Leu-DBE docks loosely to the shallower P2 pocket of BtCDPS_intermediate_. The less favourable productive docking poses to BtCDPS_intermediate_ place the leucine moiety close to the catalytic residues and the DBE moiety on the periphery of the P2 pocket. These docking experiments support our hypothesis of an unfavourable second catalytic step. If this was the case, a trapped acyl–enzyme intermediate should be formed during the catalytic cycle.

### Intact protein mass spectrometry

**Fig. 5:**
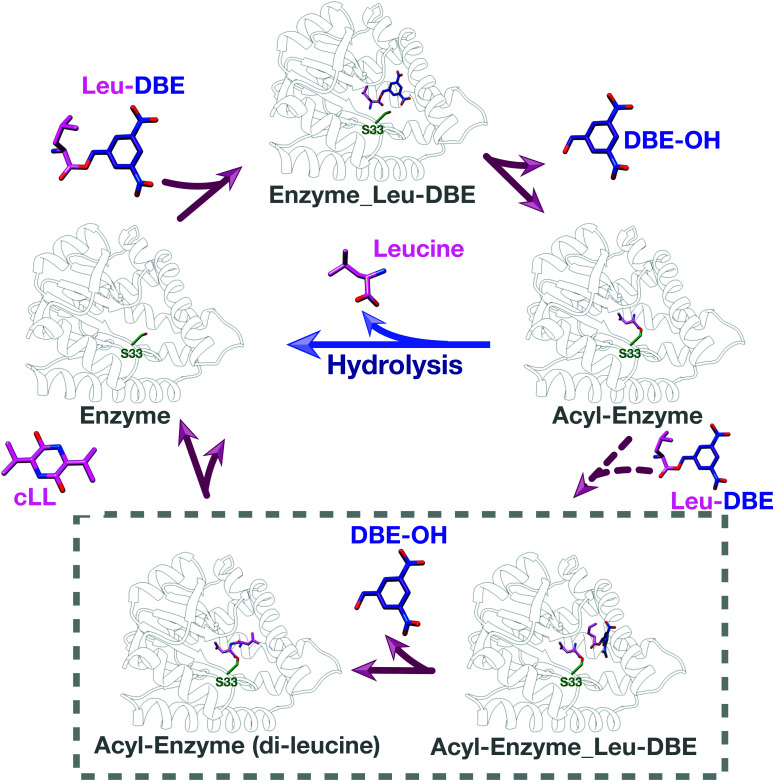
Catalytic cycle when Leu-DBE is used as a substrate. Individual panels were generated using a combination of experimental data, docking results, and modelling based on a covalent intermediate bound to AlbC (pdb 4q24).

We used intact protein mass-spectrometry to analyse the state of the protein after incubation with Leu-DBE. As we hypothesized, an accumulation of the enzyme species with a mass increase of +115 was detected, which corresponded to a trapped acyl intermediate (Fig. 3F). This result suggests that after completion of the first half reaction the enzyme becomes catalytically trapped, as substrate positioning on P2 is deficient when DBE-Leu is used as a substrate (Fig. 5). A similar mass shift was not observed when inactive mutant S33A or catalytic residue S33C variants were tested (Fig. S16, ESI†). A similar mass was also absent when the reaction was conducted with a full length aa-tRNA substrate (Fig. S17, ESI†).

These data combined suggest a role were the tRNA group of the aa-tRNA substrate acts to efficiently position the amino acid moiety in the binding pockets and restrict how many conformations can be sampled by a substrate molecule. Consequently, smaller substrates such as Leu-DBE are able to sample multiple productive and unproductive conformations, especially when interacting with the more open shallow P2 pocket.

## Conclusions

Our work demonstrates that BtCDPS is selective for the amino acid but less selective towards the tRNA portion of aa-tRNA substrates. We show, for the first time, that a CDPS can use a small molecule as a substrate to synthesise cyclic dipeptide products rather than a conventional aa-tRNA or shorter RNAs. Bypassing the need to generate aa-tRNA substrates for CDPS enzymes provides an additional tool in generating cyclic dipeptide products, not restricted by the substrate tolerance of tRNA synthetases. Future work should now be focussed on generating more efficient and stable small molecule substrates and enzyme variants. Our work provides insights into the structure of a thermophilic CDPS enzyme and identifies key residues involved in substrate recognition. Alongside identifying a minimal substrate (albeit less efficient than natural aa-tRNA), this is a perfect informed starting point for enzyme engineering, with the intention to produce more diverse products, in an efficient simply manner. The main requirement for successful protein design and *in vitro* evolution is sufficient measurable activity, allowing activity screens to be performed with enzyme variants, a condition fulfilled by Leu-DBE and BtCDPS.^39^

In a broader sense, our work highlights the fact that throughout evolution aa-tRNA-dependent enzymes adapted to utilize energetically expensive full length aa-tRNAs as substrates as these were the molecules containing activated amino acids amply available in the cell, without relying on non-ribosomal peptide synthesis machineries.^3,40^ However, other unnatural substrates can be used as alternatives, bypassing the requirement of a full length tRNA. A similar situation was observed in aminoacyl protein transferases, which transfer amino acids to other proteins and can use small molecules as aa-tRNA mimics.^41^ Here we set the foundation to developing minimalistic aa-tRNA mimics for CDPS enzymes.

## Experimental

### General

Unless mentioned, all chemicals were from Sigma or Fisher Scientific, all experiments were performed in triplicate, and all error is reported as standard deviation.

### Cloning, expression and purification

The CDPS gene from *Bacillus thermoamylovorans* (BtCDPS) was synthesised as a codon optimised gBlock from IDT. The synthesised gene was inserted into a modified pJ414 expression plasmid (TEV-cleavable His_6_ protein tag) by Gibson assembly^42^ cloning technique. Mutant variants of BtCDPS were produced by site-directed mutagenesis based on the NEB Q5 site-directed mutagenesis kit. Constructs were confirmed by sequencing and introduced into the commercially available *E. coli* Bl21(DE3) expression strain (NEB). Cells were cultured at 37 °C until the OD_600_ reached 0.6; at which time protein production was induced using 1 mM IPTG; the temperature was lowered to 18 °C and the cells continued to grow overnight.

Cell pellets were resuspended with appropriate amounts of lysis buffer (50 mM HEPES pH 7.5, 20 mM imidazole, 250 mM NaCl) and lysed using sonication, with centrifugation at 33 000*g* (4 °C) for 30 minutes to clarify the lysate. The cell lysate was passed through an 80 μm filter before being loaded onto a 5 ml HisTrap HP columns (GE Healthcare) pre-equilibrated in lysis buffer. Columns were washed with ∼20 column volumes of lysis buffer and protein eluted using elution buffer (50 mM HEPES pH 7.5, 250 mM NaCl, 400 mM imidazole). TEV protease was added (1 : 25 ratio) to pooled elution fractions and the sample dialysed overnight at 4 °C (in 20 mM HEPES pH 7.5, 250 mM NaCl, 5 mM β-mercaptoethanol). Cleaved protein was separated from residual fusion protein by a second passage over the HisTrap column. The resulting purified protein was concentrated to ∼10 mg ml^−1^ for further experiments.

#### Structure determination

Crystals were grown at 20 °C using sitting drop vapour diffusion technique with drops composed of equal volumes of protein and reservoir solution. BtCDPS crystals were grown in 0.1 M Na Citrate pH 5.5, 37.5% PEG 550 MME, 5% 1,2-hexanediol. Crystals were cryoprotected in mother liquor supplemented with 20% (v/v) ethylene glycol before being flash cooled in liquid nitrogen. Diffraction data was collected at the Diamond Light Source in Oxford, UK. Data reduction and processing was completed using XDS and the *xia2* suite.^43^ The structure was solved by molecular replacement with PHASER^44^ using the structure of yvmC-BLIC as a search model (PDB: 3OQH). The other crystal forms (BtCDPS_S33C_ and BtCDPS_R153A_) were solved using the structure of WT BtCDPS as the search model in PHASER.^44^ Protein structures were built/modified using COOT,^45^ with cycles of refinement in PHENIX^46^ and PDB-REDO.^47^ Crystallographic data is shown in Table S3 (ESI†).

### tRNA preparation

tRNA was prepared by an *in vitro* transcription reaction similarly to that described in.^48^ Template DNA of tRNA^Leu^ genes from *Bacillus thermoamylovorans* were first amplified by PCR using a set of primers, which included a T7 RNA promoter sequence (Table X, ESI†). Briefly, the *In vitro* transcription reaction was performed by incubating (at 37 °C for 12 hours) the following reaction mixture: 20 μg template DNA, 20 mM MgCl_2_, 50 mM HEPES pH 7.5, 2 mM Spermidine, 20 mM DTT, 5 mM ATP, 5 mM UTP, 5 mM CTP, 6 mM GTP, 5 μM RNA polymerase Δ172–173^49^ (to ensure tRNA homogeneous 3′ends). An appropriate amount of RNAse free DNAseI was added as per manufactures recommendations (Promega) and incubated for 1 hour at 37 °C. After incubation, samples were ran on a urea-TBE-PAGE gel to verify tRNA production. A phenol-chloroform extraction followed by ethanol precipitation was used to clean up tRNA. The tRNA was resuspended in DEPC treated water and the concentration was measured calculated from their A260 readings.

### Cyclodipeptide-synthesizing activity assay

The CDPS activity of BtCDPS and variants was measured with a coupled end-point assay, containing aaRSs (tRNA synthetase) to maintain a continuous supply aa-tRNA substrate. The assay was performed in a buffer containing 100 mM HEPES pH 7.5, 100 mM KCl, 20 mM MgCl_2_, 10 mM DTT, 5 mM ATP, 1 mM l-Leucine, 5 μM Leucine tRNA synthetase (LeuRS), 10 μM BtCDPS, 5 μM tRNA^Leu^. The tRNA isoacceptors (TTG, TTA, CTA, CTC, CTG) were assayed independently. The reaction was carried out at 20 °C whilst mixing end-over-end. The enzymatic reaction was initiated by the addition of BtCDPS and quenched by the addition of an equal volume of 10% trichloroacetic acid (TCA) and immediately boiled for 5 minutes. The samples were then prepared for analysis by LC–MS (waters) by centrifugation at 12 000*g* for 10 minutes to remove insoluble material, and supernatant was used for analysis.

A time-course assay (using the same reaction mix as previous stated) was used to assess cLL formation over a period of time. The reaction was initiated by addition of 1 μM BtCDPS, and immediately mixed well. The reaction was carried out at 20 °C, with no further mixing. 20 μl of reaction was removed at specific time intervals (10, 20, 40, 60 and 90 minutes) and quenched by the addition of an equal volume of 10% trichloroacetic acid (TCA) and immediately boiled for 5 minutes. Samples were prepared for mass spectrometry analysis as previous stated.

Assays conducted to assess BtCDPS ability to utilise misaminoacylated tRNA substrates were performed following the same procedure as for the standard cyclodipeptide-synthesizing activity assay with minor adjustments. LeuRS-D345A or Ile-D342A mutants (lacking editing activity) were created by site directed mutagenesis and used in instead of LeuRS. Assays used LeuRS-D345A or Ile-D342A paired with tRNA^Leu^ (isoacceptor mix – TTG, TTA, CTA, CTC, CTG) and tRNA^Ile^ (isoacceptor – ATC), respectively. Assays used 10 mM of either l-leucine, l-isoleucine, l-methionine or l-valine. Appropriate controls lacking BtCDPS or tRNA were conducted in parallel.

### Leu-DBE assay

Leu-DBE was chemically synthesised in house (see chemical synthesis section in the ESI†). Leu-DBE was tested as a substrate for BtCDPS in an endpoint assay. The assay was initiated by adding 5 μM BtCDPS to a reaction mixture containing 50 mM HEPES pH 7.5, 500 mM NaCl, 5 mM DTT, 500 μM Leu-DBE. The assay was incubated at room temperature and agitated by end-over-end rotation over-night. The reaction was quenched by the addition of an equal volume of 10% TCA and boiled for 5 minutes. The precipitant was removed by centrifugation at 12 000*g* for 10 minutes to remove insoluble material, and supernatant was transferred to vials. The samples were then used for analysis by LC-MS (orbitrap) or HPLC as described below.

A Leu-DBE time-course assay, monitoring formation of cLL at regular 40 minute timepoints over the period 0–280 minutes (& 24 hours), to coincide with the Leu-DBE hydrolysis, HPLC assay (see HPLC section). Samples were quenched by the addition of TCA (100%) to a final concentration of 10% TCA. Quenched samples were centrifuged to removed insoluble material and diluted 50% v/v with water. Samples were analysed using LC-MS (orbitrap).

Met-DBE and Ile-DBE were used as substrates instead of Leu-DBE in the Leu-DBE assay to test if alternative amino acids coupled to DBE could be used as substrates. Experiments ± 1 mM Met-DBE or Ile-DBE were incubated at 20 °C for 24 hours. The reactions were quenched and prepared as stated previously for Leu-DBE assay samples.

### Cyclodipeptide detection by LC-MS

Analysis of samples using tRNA substrates was performed using a Waters Acquity UPLC H class plus coupled to a Xevo G2-XS QTof Quadrupole Time-of-Flight mass spectrometer. Samples were loaded onto a Waters, ACQUITY UPLC BEH C18 Column (130 Å, 1.7 μm, 2.1 mm × 50 mm).

The LC program was as follows: 0–1 min= 1% D, 1–6 min = linear gradient from 1–99% D, 6–9 min = 99% D, 9–12 min = D, with a 0.4 ml min^−1^ flow rate (D = (v/v) acetonitrile in 0.1% FA).

Data collected using the MS^e^ mode, the mass spectrometer was set in the electrospray positive ion mode to detect *m*/*z* in the 100–700 Da range (scan time 0.1 s, ramp collision energy = 15–30 V, dynamic range = normal, method time = 0–12 min). A lockspray correction by collecting every 10 s, averaging 3 scans of 1 s each using Leucine Enkephalin (LeuEnk) as standard (556.2771 ± 0.56 Da). Cyclodipeptides were quantified based on the peak area for the expected extracted mass. A cLL standard, prepared by over-production and purification of cLL by HPLC, was used to produce a calibration curve.

Samples from Leu-DBE, Met-DBE and Ile-DBE assays were analysed using a Thermo Scientific Orbitrap Velos Pro with U3000 HPLC, with a Phenomenex Luna omega C18 column (100 Å, 1.6 μm, 2.1 mm × 50 mm). The LC program was as follows: 0–5 min = 2% D, 5–10 min = linear gradient from 2–70% D, 10–10.5 min = linear gradient from 70–99% D, 10.5–13 min = 99% D, 13–13.1 min = 2% D, 13.1–15 min = 2% D, with a flow rate of 0.2 ml min^−1^ (D = (v/v) acetonitrile in 0.1% FA).

MS data collected throughout the 15 min LC run with the following settings: polarity = positive, analyzer = FTMS, mass range = normal, resolution = 15 000, scan range = 200–500 Da range *m*/*z*. Cyclodipeptides were quantified as previously stated above.

### Cyclodipeptide detection by HPLC

HPLC analysis was performed using a Shimadzu Prominence UFLC, HPLC coupled to a Shimadzu ELSD-LT II detector, to detect and collect cLL from assay samples. The samples were loaded onto a C18 column (NUCLEODUR 100-5 C18 250 × 4.6 mm), run with a linear gradient from 0.5% to 100% (v/v) acetonitrile in 0.1% (v/v) TFA, at a flow rate of 1 ml min^−1^. The program was as follows:

0–5 min, 0.5% (v/v) acetonitrile in 0.1% (v/v) TFA 5–40 min, linear gradient from 20–80% (v/v) acetonitrile in 0.1% (v/v) TFA, 40–45 min, 99% (v/v) acetonitrile in 0.1% (v/v) TFA, 45–50 min, 1% (v/v) acetonitrile in 0.1% (v/v) TFA.

Absorbance was monitored at 220 nm wavelength and with the ELSD detector (temperature 40 °C, gain 12). For extraction of cLL, peaks with a retention time of 24 min, were collected by a Shimadzu FRC-10A fraction collector. Collected peak fractions were pooled and freeze dried to remove liquid and resuspended in 50/50 water/DMSO. The sample was then checked using HRMS and quantified using the absorbance reading at 215 nm on a DeNovix DS-11 FX spectrophotometer on peptide mode (*ε*_0.1%_ = 11.7).

HPLC was also used to measure hydrolysis of Leu-DBE in the presence of different concentrations of BtCDPS. Leu-DBE and DBE-OH standards had retention times of 19.5 min and 21 min, respectively. Leu-DBE has a half-life of 431 min in our assay conditions (Fig. S20, ESI†), and therefore Leu-DBE standards were run immediately after resuspension in water to reduce the hydrolysis associated with long term storage in water.

HPLC was used to analyse hydrolysis of Leu-DBE and formation of DBE-OH from our Leu-DBE time-course assay (same reaction materials as previously stated, 50 μM of enzyme used in reaction). Hydrolysis of Leu-DBE occurs spontaneously in water, so reactions were quenched at regular 40 minute intervals from 0–280 minutes (& 24 hours), to coincide with the HPLC run time. Samples were quenched by the addition of TCA (100%) to a final concentration of 10% TCA. Quenched samples were immediately vortexed and centrifuged for 2 minutes to remove insoluble material. 50 μl of samples were immediately injected onto the HPLC.

Concentration was calculated as follows: 1 mM = (peak area corresponding to DBE-Leu + peak area corresponding to DBE-OH).

### Identification of the covalent peptidyl-enzyme intermediate

Acyl-intermediate enzyme preparation was performed by incubating 20 μM enzyme with excess Leu-DBE (500 μM or 2.5 mM), for 2 h or overnight by end-over-end rotation. The samples were then sent for analysis at the University of St Andrews mass spectrometry and proteomics facility. The method for analysis was as follows:

20 μl of sample at 1 : 20 dilution, was injected onto a Waters MassPrep micro column 2 mm × 5 mm on a Waters Xevo LC-MS system optimised for protein analysis. A short gradient elution was used to desalt and then elute the protein as follows:Time (min) Flow Rate (ml min^−1^) %A = 98% water (1%FA) 2% Acetonitrile %B = 98% acetonitrile (1%FA) 2% water.

(1) Initial 0.200 98.0 2.0.

(2) 0.50 0.200 98.0 2.0.

(3) 3.80 0.200 2.0 98.0.

(4) 4.50 0.200 2.0 98.0.

(5) 4.60 0.200 100.0 0.0.

(6) 5.00 0.200 100.0 0.0.

The MS was operated in ESI^+^ and scanned from 500–2500 *m*/*z* with lock mass of LeuEnk as described above under “Cyclodipeptide detection by LC-MS”. The protein spectrum elution at 3 minutes was combined and the raw data processes to mass using MaxEnt algorithm at 0.1 resolution using peak width of half height of 0.4 Da.

### Chemical synthesis of Leu-PANS, Leu-cou, Leu-DBE, Val-DBE, Ile-DBE and Met-DBE)

Chemical synthesis of aa-DBE compounds were carried out following the procedures described by Peacock JR, *et al.*^50^ Detailed procedures are on the ESI.† NMR was used to verify aa-DBE compounds identity and purity (Fig. S3–S7, ESI†).

Chemical synthesis of Leu-Puromycin and Leu-Coumarin were carried out following a modification of the procedures described by Stark *et al.*,^51^ and a detailed description of methods, NMR and HRMS is available on the ESI.†

### Ligand docking

The ligand Leucine-DBE was docked into the CDPS protein (PDB:6ZTU) as an apo-enzyme (CDPS_apo_) and an acyl–enzyme model (BtCDPS_intermediate_), generated by superimposition and trimming of the ligand from PDB: 4Q24 (*N*-carbobenzyloxy-l-Phe-chloromethyl ketone), using PSOVina2.^52^ First, water molecules and other heteroatoms were removed from the structure, and the program PDB2PQR 2.1.1^53^ used to assign position-optimized hydrogen atoms, utilizing the additional PropKa2 algorithm^54^ with a pH of 7.4 to predict protonation states. The MGLTools 1.5.6 utility^55^ prepare_receptor4.py was used to assign Gasteiger charges to atoms. Hydrogen atoms were assigned to compound structures using OpenBabel 2.4.1,^56^ utilizing the -p option to predict the protonation states of functional groups at pH 7.4. The MGLTools utility prepare_ligand4.py was used to assign Gasteiger charges and rotatable bonds. PSOVina2 was used to automatically dock the compounds into the second ligand-binding site of the crystal structures. A grid box that encompassed the maximum dimensions of the ligand plus 12 Å in each direction was used. The starting translation and orientation of the ligand and the torsion angles of all rotatable bonds were set to random.

## Funding statement

C. J. H. and C. M. C. are funded by the Wellcome trust (210486/Z/18/Z), ES is funded by the Cunningham trust (PhD-CT-18-41).

## Conflicts of interest

There are no conflicts to declare.

## Supplementary Material

CB-002-D0CB00142B-s001
