# Correction: Bypassing the requirement for aminoacyl-tRNA by a cyclodipeptide synthase enzyme

**DOI:** 10.1039/d1cb90009a

**Published:** 2021-04-14

**Authors:** Christopher J. Harding, Emmajay Sutherland, Jane G. Hanna, Douglas R. Houston, Clarissa M. Czekster

**Affiliations:** School of Biology, Biomedical Sciences Research Complex, University of St Andrews St Andrews Fife KY16 9ST UK cmc27@st-andrews.ac.uk; Arab Academy for Science, Technology, and Maritime Transport (AASTMT) Cairo Campus Egypt; Institute of Quantitative Biology, Biochemistry and Biotechnology, University of Edinburgh, King's Buildings Waddington 1 Building Edinburgh EH9 3BF UK

## Abstract

Correction for ‘Bypassing the requirement for aminoacyl-tRNA by a cyclodipeptide synthase enzyme’ by Christopher J. Harding *et al.*, *RSC Chem. Biol.*, 2021, **2**, 230–240, DOI: 10.1039/D0CB00142B.

The authors regret that ref. 30–49 were incorrectly shown in the original article. The corrected references are shown here.

30. N. Canu, C. Tellier, M. Babin, R. Thai, I. Ajel, J. Seguin, O. Cinquin, R. Vinck, M. Moutiez, P. Belin, J. C. Cintrat and M. Gondry, Flexizyme-aminoacylated shortened tRNAs demonstrate that only the aminoacylated acceptor arms of the two tRNA substrates are required for cyclodipeptide synthase activity, *Nucleic Acids Res.*, 2020, **48**(20), 11615–11625.

31. N. Cvetesic, J. J. Perona and I. Gruic-Sovulj, Kinetic partitioning between synthetic and editing pathways in class I aminoacyl-tRNA synthetases occurs at both pre-transfer and post-transfer hydrolytic steps, *J. Biol. Chem.*, 2012, **287**(30), 25381–25394.

32. A. C. Bishop, T. K. Nomanbhoy and P. Schimmel, Blocking site-to-site translocation of a misactivated amino acid by mutation of a class I tRNA synthetase, *Proc. Natl. Acad. Sci. U. S. A.*, 2002, **99**(2), 585–590.

33. M. C. Hartman, K. Josephson, C. W. Lin and J. W. Szostak, An expanded set of amino acid analogs for the ribosomal translation of unnatural peptides, *PLoS One*, 2007, **2**(10), e972.

34. N. Canu, P. Belin, R. Thai, I. Correia, O. Lequin, J. Seguin, M. Moutiez and M. Gondry, Incorporation of Non-canonical Amino Acids into 2,5-Diketopiperazines by Cyclodipeptide Synthases, *Angew. Chem., Int. Ed. Engl.*, 2018, **57**(12), 3118–3122.

35. I. Wohlgemuth, M. Beringer and M. V. Rodnina, Rapid peptide bond formation on isolated 50S ribosomal subunits, *EMBO Rep.*, 2006, **7**(7), 699–703.

36. K. Suto, Y. Shimizu, K. Watanabe, T. Ueda, S. Fukai, O. Nureki and K. Tomita, Crystal structures of leucyl/phenylalanyl-tRNA-protein transferase and its complex with an aminoacyl-tRNA analog, *EMBO J.*, 2006, **25**(24), 5942–5950.

37. M. Ohuchi, H. Murakami and H. Suga, The flexizyme system: a highly flexible tRNA aminoacylation tool for the translation apparatus, *Curr. Opin. Chem. Biol.*, 2007, **11**(5), 537–542.

38. H. Murakami, A. Ohta, Y. Goto, Y. Sako and H. Suga, Flexizyme as a versatile tRNA acylation catalyst and the application for translation, *Nucleic Acids Symp. Ser.*, 2006, (50), 35–36.

39. C. Zeymer and D. Hilvert, Directed Evolution of Protein Catalysts, *Annu. Rev. Biochem.*, 2018, **87**, 131–157.

40. C. S. Francklyn and A. Minajigi, tRNA as an active chemical scaffold for diverse chemical transformations, *FEBS Lett.*, 2010, **584**(2), 366–375.

41. A. M. Wagner, M. W. Fegley, J. B. Warner, C. L. Grindley, N. P. Marotta and E. J. Petersson, N-terminal protein modification using simple aminoacyl transferase substrates, *J. Am. Chem. Soc.*, 2011, **133**(38), 15139–15147.

42. D. G. Gibson, Synthesis of DNA fragments in yeast by one-step assembly of overlapping oligonucleotides, *Nucleic Acids Res.*, 2009, **37**(20), 6984–6990.

43. G. Winter, xia2: an expert system for macromolecular crystallography data reduction, *J. Appl. Crystallogr.*, 2010, **43**, 186–190.

44. A. J. McCoy, R. W. Grosse-Kunstleve, P. D. Adams, M. D. Winn, L. C. Storoni and R. J. Read, Phaser crystallographic software, *J. Appl. Crystallogr.*, 2007, **40**, 658–674.

45. P. Emsley, B. Lohkamp, W. G. Scott and K. Cowtan, Features and development of Coot, *Acta Crystallogr., Sect. D: Biol. Crystallogr.*, 2010, **66**, 486–501.

46. P. V. Afonine, R. W. Grosse-Kunstleve, N. Echols, J. J. Headd, N. W. Moriarty, M. Mustyakimov, T. C. Terwilliger, A. Urzhumtsev, P. H. Zwart and P. D. Adams, Towards automated crystallographic structure refinement with phenix.refine., *Acta Crystallogr., Sect. D: Struct. Biol.*, 2012, **68**, 352–367.

47. R. P. Joosten, F. Long, G. N. Murshudov and A. Perrakis, The PDB_REDO server for macromolecular structure model optimization, *Iucrj*, 2014, **1**, 213–220.

48. B. Beckert and B. Masquida, Synthesis of RNA by *In Vitro* Transcription, *Rna: Methods Protoc.*, 2011, **703**, 29–41.

49. D. L. Liakhov, K. Il'genfrits, B. K. Chernov, S. M. Dragan, V. O. Rechinskii, D. K. Pokholok, V. L. Tunitskaia and S. N. Kochetkov, Site-specific mutagenesis of residue Lys-172 of phage T7 RNA polymerase: characterization of transcription properties of mutant proteins, *Mol. Biol.*, 1992, **26**(5), 1022–1035.

The Royal Society of Chemistry apologises for these errors and any consequent inconvenience to authors and readers.

## Supplementary Material

