# Supplementary material for: Bypassing the requirement for aminoacyl-tRNA by a cyclodipeptide synthase enzyme
Source: RSC Chem Biol. 2021 Jan 15;2(1):230–40. doi: 10.1039/d0cb00142b (PMC8084100; doi:10.1039/d0cb00142b)
Supplement: CB-002-D0CB00142B-s001 [file CB-002-D0CB00142B-s001.pdf]

Electronic Supplementary Material (ESI) for RSC Chemical Biology.
This journal is © The Royal Society of Chemistry 2021

Supporting information for

Bypassing the requirement for aminoacyl-tRNA by a cyclodipeptide synthase enzyme

Christopher J. Harding,[a] Emmajay Sutherland,[a] Jane Hanna,[b] Douglas Houston,[c] Clarissa M. Czekster,[a]

Index

Supplementary methods – Melting curve for BtCDPS	2
Supplementary methods – ITC	2
Supplementary methods – Leu-PANS inhibition	2
Supplementary methods – Instrumentation and general	2
Supplementary methods – chemical synthesis of *Leu-UMB (**2**)*	3
Supplementary methods – chemical synthesis of Leu-PANS (**3**)	4
Supplementary methods – chemical synthesis of aa-*DBE compounds*	5
Supporting Figure 1 - *^{1}H NMR spectra of Leu-UMB (**2**)*	4
Supporting Figure 2 - *HRMS spectra of Leu-PANS (**3**)*	4
Supporting Figure 3 - *^{1}H NMR spectra of Leu-DBE (**4**)*	6
Supporting Figure 4 - *^{1}H NMR spectra of Pro-DBE (**5**)*	7
Supporting Figure 5 - *^{1}H NMR spectra of Val-DBE (**6**)*	7
Supporting Figure 6 - *^{1}H NMR spectra of Ile-DBE (**7**)*	8
Supporting Figure 7 - *^{1}H NMR spectra of Met-DBE (**8**)*	8
Supporting Figure 8 - *Melting curve for BtCDPS*	9
Supporting Figure 9 - *HRMS spectra of unnatural aa-containing cyclic dipeptides*	10
Supporting Figure 10 - *tRNA misaminoacylation experiment*	11
Supporting Figure 11 - *Alternative aa-DBE substrates*	12
Supporting Figure 12 - *Possible role of Arg153*	13
Supporting Figure 13 - *BtCDPS mutant structures*	14
Supporting Figure 14 - *Sequence alignment of characterised CDPS*	15
Supporting Figure 15 - *Sequence alignment of tRNA^{Leu} from B. thermoamylovorans*	16
Supporting Figure 16 - *Intact mass spec of S33A and S33C reaction with DBE-Leu*	17
Supporting Figure 17 - *Intact mass spec of BtCDPS - tRNA assay*	18
Supporting Figure 18 - *Comparison between minimal substrates*	19
Supporting Figure 19 - ITC data for Leu-PANS	20
Supporting Figure 20 - *Leu-DBE hydrolysis with and without BtCDPS*	21
Supporting Figure 21 - *tRNA assay timecouse, Leu-PANS inhibition, R153A activity*	22
Supporting Table 1 - *HPLC conditions for purifying compound*	4
Supporting Table 2 - *Absorbance and concentration of Leu-PANS (**3**)*	5
Supporting Table 3 - *Crystallographic data*	23
Supporting References	24

Supporting Materials and Methods

Melting curve for BtCDPS

A melting curve experiment was performed on BtCDPS in storage buffer, with 4 independent replicates (n=4). The sample mixture (50 µl), included: 1 µM BtCDPS, 2x Sypro Orange, 20 mM HEPES pH 7.5, 250 mM NaCl, 5 mM β-mercaptoethanol, final concentrations. Samples were mixed well and loaded into a 96 well plate and centrifuged for 1 minute to remove air bubbles. The samples were exposed to a ramped heat change of 1 °C every minute from 25 °C to 95 °C using a MX3000P qPCR Machine from Stratagene. The instrument was configured to use the SYPRO FRROX filter set, excitation at 492 and emission at 610 nm with x4 gain setting.

ITC

ITC experiments were performed using a Microcal PEAQ-ITC. The experiments were performed using the following settings: Temperature = 25°C, Reference Power = 10, Feedback = high, Stir speed = 500, Initial delay = 60s, 19 injections (1 injection of 0.5 µl, for 0.8 s and 18 injections of 2 µl for 4s with a spacing of 120 s between injections). The experiments were performed in a binding buffer, 100mM HEPES pH 7.5, 500 mM NaCL and 1 mM DTT. BtCPDS and Leu-PANS were buffer exchanged into binding buffer immediate prior to performing the binding experiment.

Leu-PANS inhibition assay

The Leu-PANS inhibition assay was performed similarly to the tRNA timecourse assay. The coupled end-point assay, contained a mixture of leucyl-tRNA synthetass to maintain a continuous supply of Leu-$tRNA^{Leu}$ substrate. The assay was performed in a buffer containing 100 mM HEPES pH 7.5, 100 mM KCl, 20 mM $MgCl_2$, 10 mM DTT, 5 mM ATP, 1 mM L-Leucine, 5 µM Leucine tRNA synthetase (LeuRS), 5 µM $tRNA^{Leu}$ (mixture of isoacceptors). The reaction was initaiated by addition of 1 µM BtCDPS, and immediately mixed well. The reaction was carried out at 20 °C, with no further mixing. 20 µl of reaction was removed at specific time intervals (20, 40, 60 and 90 minutes) and quenched by the addition of an equal volume of 10% trichloroacetic acid (TCA) and immediately boiled for 5 minutes. The samples were then prepared for analysis by LC–MS by centrifugation at 12000g for 10 minutes to remove insoluble material, and supernatant was used for analysis. The assay was performed in the presence of either 0 mM, 0.5 mM and 2 mM Leu-PANS (dissolved in H_2O). Triplicate experiments were carried out for each Leu-PANS concentration.

Instrumentation and General Methods

All chemical compounds and reagents were purchased from various commercial sources and were used without further purification. All non-aqueous reactions were run under an atmosphere of dried argon in dried glassware, and moisture sensitive reagents were added through a syringe. Analytical thin-layer chromatography (TLC) was performed using 0.25 mm silica gel 254-F plates and visualisation was accomplished with UV light. Flash column chromatography was packed with Silica Gel 60. High performance liquid chromatography (HPLC) was performed using a Shimadzu UFLC system coupled to a UV-vis detector equipped with a C18 column (EC HPLC Nucleodur, Macherey Nagel, 3 µm particle size, 250 x 4.6 mm). NMR spectra was recorded on a Bruker AV 400 MHz spectrometer with a BBFO probe. Chemical shifts are reported in parts per million (ppm) and the data was detailed as follows:

chemical shift, multiplicity (s = singlet, d = doublet, t = triplet, q = quartet, m = multiplet), coupling constants, and number of protons.

Synthetic Methods

*Synthesis of 4-Methyl-2-oxo-2H-chromen-7-yl N-{[(2-methyl-2-propanyl)oxy]carbonyl}-D-leucinate (**1**)*

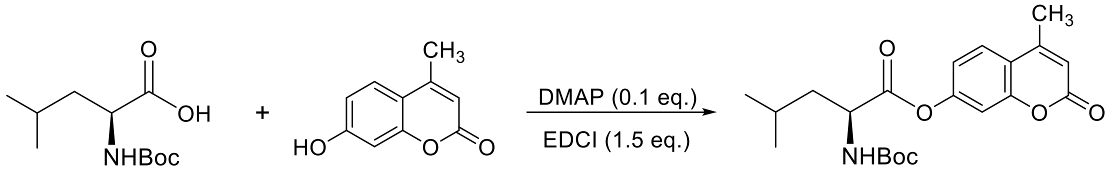

Boc-L-leucine 4-methylumbelliferone **1**

Boc = 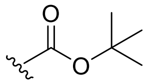

A mixture of boc-L-leucine (382.0 mg, 1.65 mmol, 1 eq.), 4-methylumbelliferone (362.8 mg, 2.06 mmol, 1.25 eq.), N-(3-Dimethylaminopropyl)-N′-ethylcarbodiimide hydrochloride (EDCI, 476.8 mg, 2.49, 1.5 eq.) and 4-dimethylaminopyridine (DMAP, 26 mg, 0.213 mmol, 0.1 eq.) in dichloromethane was stirred at room temperature under argon overnight. The reaction was monitored via thin layer chromatography (TLC, EtOAc:Hexane (40:60), R_f 0.60) and visualised under UV. An attempt to remove the residual 4-methylumbelliferone by washing with water was unsuccessful therefore product **1** was purified by silica gel column chromatography using EtOAc:Hexane (40:60) as the eluent (452.8 mg, 70% yield). ^{1}H NMR (400 MHz, Chloroform-d) δ 7.61 (d, J = 8.6 Hz, 1H), 7.15 – 7.05 (m, 2H), 6.27 (q, J = 1.3 Hz, 1H), 4.94 (d, J = 8.4 Hz, 1H), 4.53 (s, 1H), 2.43 (d, J = 1.3 Hz, 3H), 2.04 (s, 1H), 1.88 – 1.73 (m, 2H), 1.73 – 1.61 (m, 1H), 1.46 (s, 9H), 1.25 (t, J = 7.1 Hz, 1H), 1.02 (dd, J = 6.3, 2.0 Hz, 6H).

*Synthesis of 4-Methyl-2-oxo-2H-chromen-7-yl D-leucinate (Leu-UMB (**2**))*

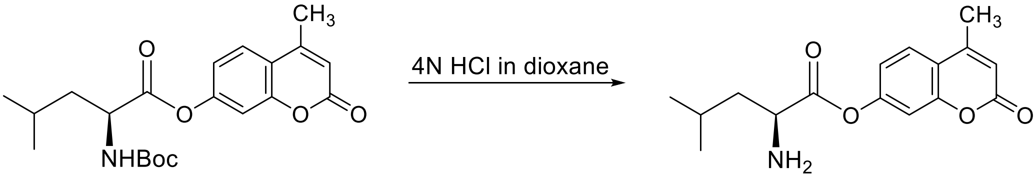

1 Leu-UMB (**2**)

To a flask flushed with argon, dried product **1** (102.2 mg, 0.262 mmol) was dissolved in 4N HCl in dioxane (2 mL). This solution was stirred at room temperature for 30 minutes and the reaction was monitored via TLC. The reaction was stopped when a white precipitate had formed, and the spot related to the boc -protected product **1** had disappeared. This solid was recovered via vacuum filtration and washed with diethyl acetate (2 x 5 mL) to give the unprotected species, **2** (66.7 mg, 88% yield). ^{1}H NMR (400 MHz, Methanol-d_4) δ 7.90 (d, J = 8.7 Hz, 1H), 7.31 (d, J = 2.2 Hz, 1H), 7.27 (dd, J = 8.7, 2.3 Hz, 1H), 6.40 (q, J = 1.3 Hz, 1H), 4.41 (t, J = 7.0 Hz, 1H), 2.53 (d, J = 1.3 Hz, 3H), 2.12 – 2.01 (m, 1H), 2.01 – 1.83 (m, 2H), 1.11 (dd, J = 7.8, 6.3 Hz, 6H).

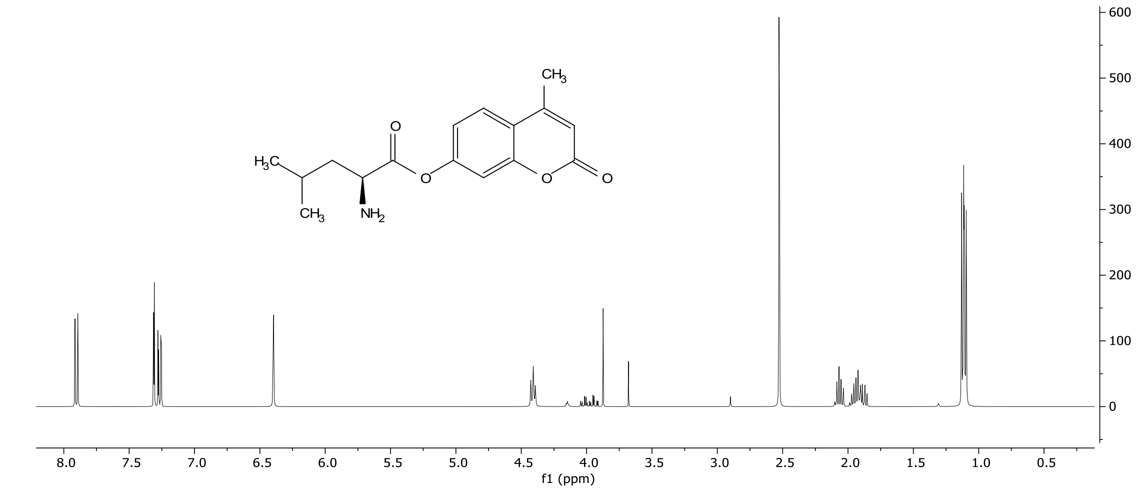

*Fig. S1 – ^{1}H NMR spectra of Leu-UMB (**2**)*

*Synthesis of (S)-2-amino-N-((2S,3S,4R,5R)-5-(6-(dimethylamino)-9H-purin-9-yl)-4-hydroxy-2-(hydroxymethyl)tetrahydrofuran-3-yl)-4-methylpentanamide[1, 2](Leu-PANS (**3**))*

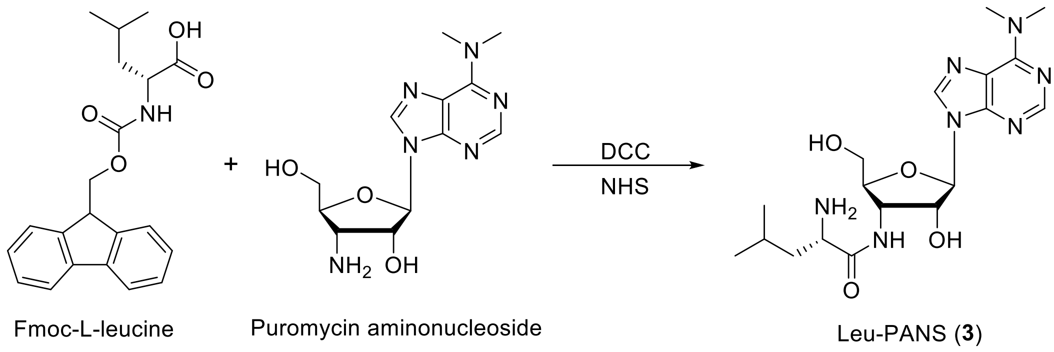

N,N'-dicyclohexylcarbodiimide (DCC, 11.9 mg, 0.0539 mmol) was added to a cold solution of puromycin aminonucleoside (15.3 mg, 0.052 mmol), fmoc-L-leucine (19.3 mg, 0.0541), and *N*-hydroxysuccinimide (NHS, 8.1 mg, 0.0556 mmol) in dried DMF (0.9 mL). This solution was stirred at 4 °C for 30 minutes followed by 4 days at room temperature whereupon a white solid had precipitated. The precipitate was removed and product **3** was separated and purified via HPLC. The subsequent fractions containing Leu-PANS were collected, dried *in vacuo* and the resultant solid was dissolved in water (0.2 mL). The concentration of the product was determined using a Nanodrop spectrophotometer at 269 nm in relation to puromycin aminonucleoside.

**Time / min**	**Solvent B (%)**
Initial	5
5.00	5
22.00	34
22.50	95
27.50	95
28.00	5
40.00	5

*Table S1 - HPLC conditions for purifying compound **3**: 1 mL/min flow rate using mobile phase A: H_2O + 0.1% trifluoroacetic acid and mobile phase B: 100% acetonitrile at 40 °C column oven. Multiple injections of 50 µL were subjected to the above run.*

Molecule	Absorbance at 269 nm	Concentration / mM
PANS	6.21	0.578
**3**	77.83	7.244

*Table S2 - Concentration of product **3** calculated by initially measuring the absorbance of a known PANS concentration and comparing the absorbance of product **3**.*

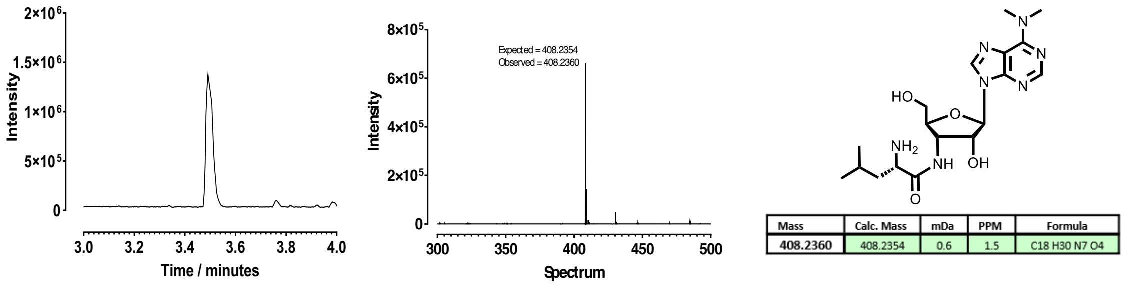

Fig S2. LC-MS of Leu-PANS analysed on an Atlantis Premier C18 AX column (Waters Acquity) using a gradient of 1% acetonitrile and 0.1% formic acid to 99% acetonitrile and 0.1% formic acid over 15 minutes.

Synthesis of DBE Amino Esters **–** *Leu-DBE, Pro-DBE, Val-DBE, Ile-DBE, Met-DBE (**4-8**) (Methods were adapted from [3])*

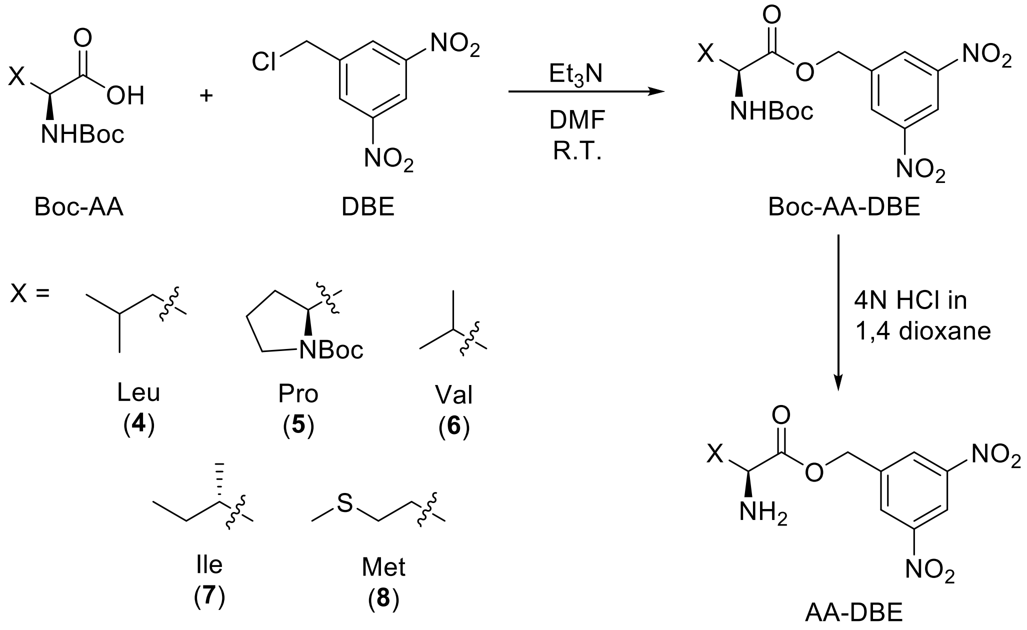

Synthesis of Boc-AA-DBE

N-Boc-L-amino acid was added to a 10 mL flask (Boc-Leu = 346.9 mg, 1.5 mmol, Boc-Pro = 322.9 mg, 1.5 mmol, Boc-Val = 395 mg, 1.5 mmol) with 3,5-dinitrobenzyl chloride (409.3 mg, 1.5 mmol). The reaction was placed under argon and DMF (1.6 mL for Leu, 2mL for Pro and Val) was added, forming a golden solution. Addition of Et_3N (0.417 mL, 2.52 mmol) quickly

gave rise to a dark red slurry, and the reaction was stirred under argon at RT and monitored by TLC (with Hexane/EtOAc 4:1 for Boc-Leu and DBE compounds). After 16 h, the reaction mixture was diluted with diethyl ether (100 mL) and washed with 1M HCl (3 x 100 mL), sat aq $NaHCO_3$ (sodium bicarbonate, 3 x 100 mL), and brine (1 x 100 mL). The organic layer was dried over Na_2SO_4, filtered, and concentrated *in vacuo* to provide the crude product as a tan solid that was used without further purification. Yields before Boc deprotection were 314.4 mg (51%, Boc-Leu-DBE), 346.5 mg (58%, Boc-Pro-DBE), 482.4 mg (81%, Boc-Val-DBE).

*Boc deprotection of AA-DBE (**4-6**)*

Crude *N*-Boc-amino acid-3,5-dinitrobenzyl ester (AA-DBE) was added to a 25 mL flask and placed under argon. 4 N HCl in 1,4-dioxane (5 mL) was added, and the resulting solution was stirred at rt for 30 min, after which deprotection was verified by TLC, and the solvent removed *in vacuo*. The resulting oil was triturated with Et_2O to provide the title compound.

*Leu-DBE (**4**)*

Off white powder, yield: 311.2 mg, 131%. ^{1}H NMR (400 MHz, DMSO-d_6) δ 8.82 (t, *J* = 2.1 Hz, 1H), 8.73 (d, *J* = 2.1 Hz, 2H), 8.53 (s, 3H), 5.50 (t, *J* = 1.1 Hz, 2H), 4.12 (t, *J* = 7.0 Hz, 1H), 3.56 (s, 1H), 1.83 – 1.59 (m, 2H), 0.90 (dd, *J* = 6.3, 3.2 Hz, 5H).

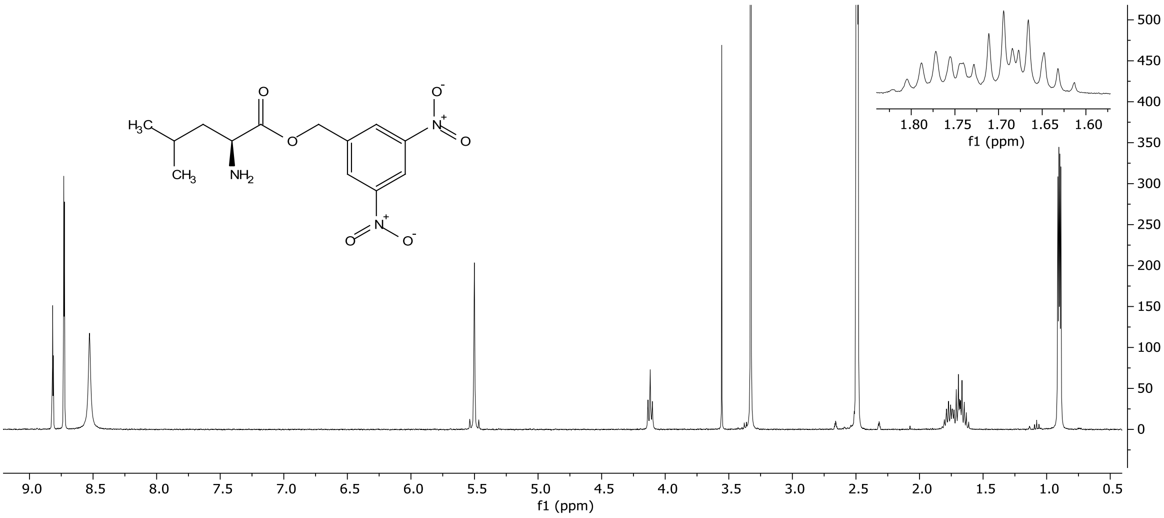

*Fig. S3 – ^{1}H NMR spectra of Leu-DBE (**4**)*

*Pro-DBE (**5**)*

White powder, yield: 132.6 mg, 51%.^{1}H NMR (400 MHz, DMSO-d$_6$) δ 8.82 (t, J = 2.2 Hz, 1H), 8.75 (d, J = 2.1 Hz, 2H), 5.49 (s, 2H), 4.51 (dd, J = 8.6, 7.3 Hz, 1H), 3.31 – 3.14 (m, 2H), 2.28 (ddd, J = 13.1, 8.5, 6.5 Hz, 1H), 2.07 (dq, J = 12.9, 7.3 Hz, 1H), 1.92 (p, J = 7.2 Hz, 2H).

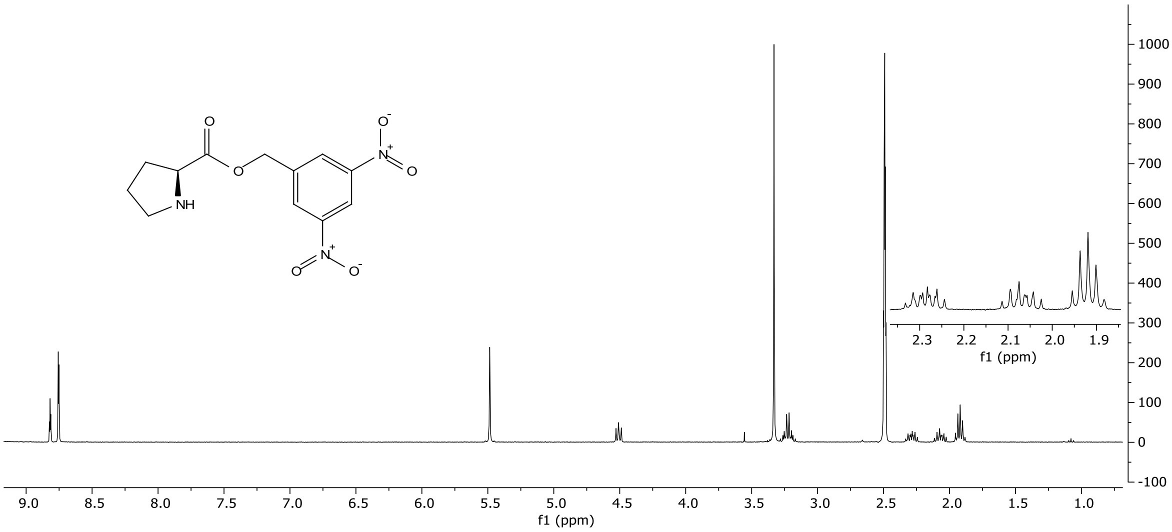

*Fig. S4 – ^{1}H NMR spectra of Pro-DBE (**5**)*

*Val-DBE (**6**)*

White powder, yield: 308 mg, 85%. ^{1}H NMR (400 MHz, DMSO-d_6) δ 8.83 (t, *J* = 2.1 Hz, 1H), 8.75 (d, *J* = 2.1 Hz, 2H), 8.55 (s, 3H), 5.53 (s, 2H), 4.05 (d, *J* = 4.7 Hz, 1H), 2.28 – 2.18 (m, 1H), 0.98 (dd, *J* = 10.8, 6.9 Hz, 6H).

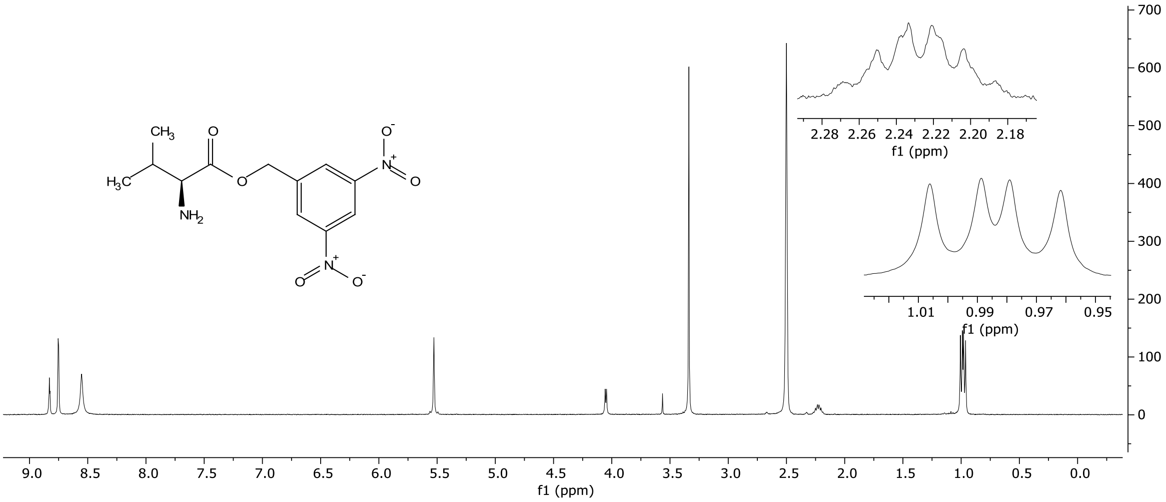

*Fig. S5 – ^{1}H NMR spectra of Val-DBE (**6**)*

*Ile-DBE (**7**)*

White powder, yield: 0.131 g, 28% over 2 steps. ^{1}H NMR (400 MHz, Methanol-δ_4): δ 8.99 (t, J = 2.1 Hz, 1H), 8.72 (dd, J = 2.1, 0.6 Hz, 2H), 5.55 (s, 2H), 4.18 (d, J = 4.0 Hz, 1H), 1.54 (dtd, J = 14.8, 7.4, 5.4 Hz, 1H), 1.46 – 1.30 (m, 2H), 1.05 (d, J = 7.0 Hz, 3H), 1.00 (t, J = 7.4 Hz, 3H).

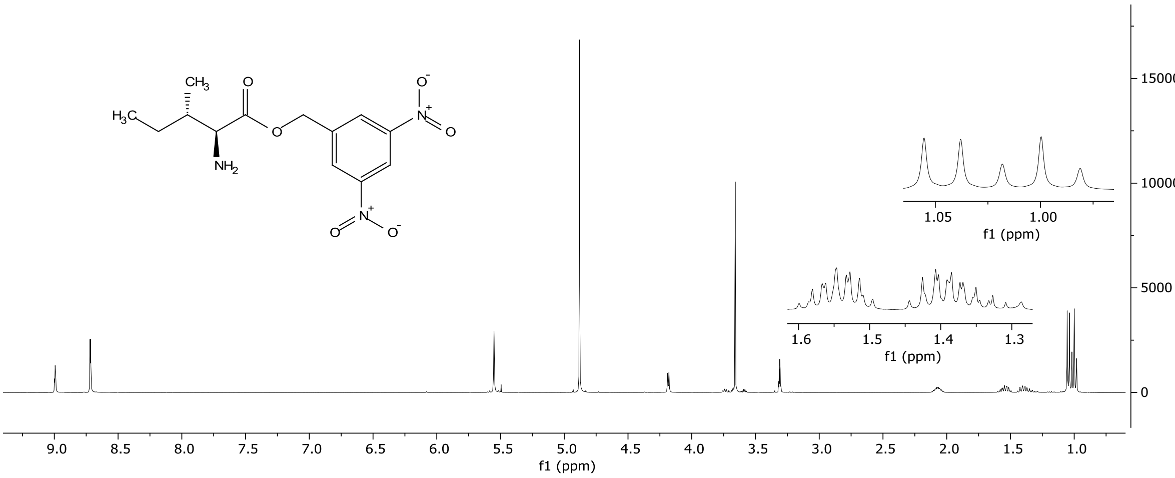

*Fig. S6 – ^{1}H NMR spectra of Ile-DBE (**7**)*

*Met-DBE (**8**)*

Orange oil, yield: 0.8013g, 59%. ^{1}H NMR (400 MHz, Methanol-d4) δ 8.92 (t, J = 2.1 Hz, 1H), 8.67 (d, J = 2.1 Hz, 1H), 5.49 (dd, J = 12.6, 5.0 Hz, 1H), 4.32 (t, J = 6.3 Hz, 1H), 2.63 (td, J = 7.3, 3.1 Hz, 1H), 2.31 – 2.12 (m, 1H), 2.04 (s, 2H).

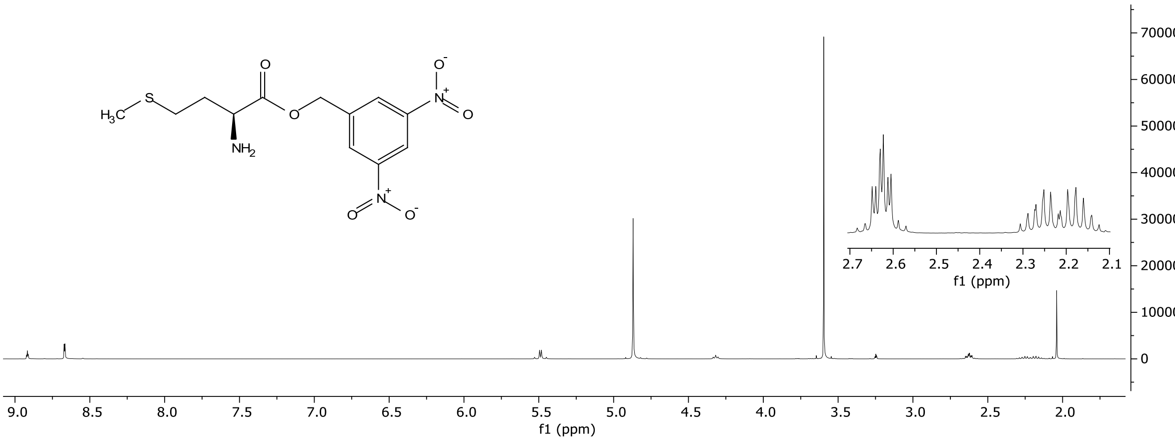

*Fig. S7 – ^{1}H NMR spectra of Met-DBE (**8**)*

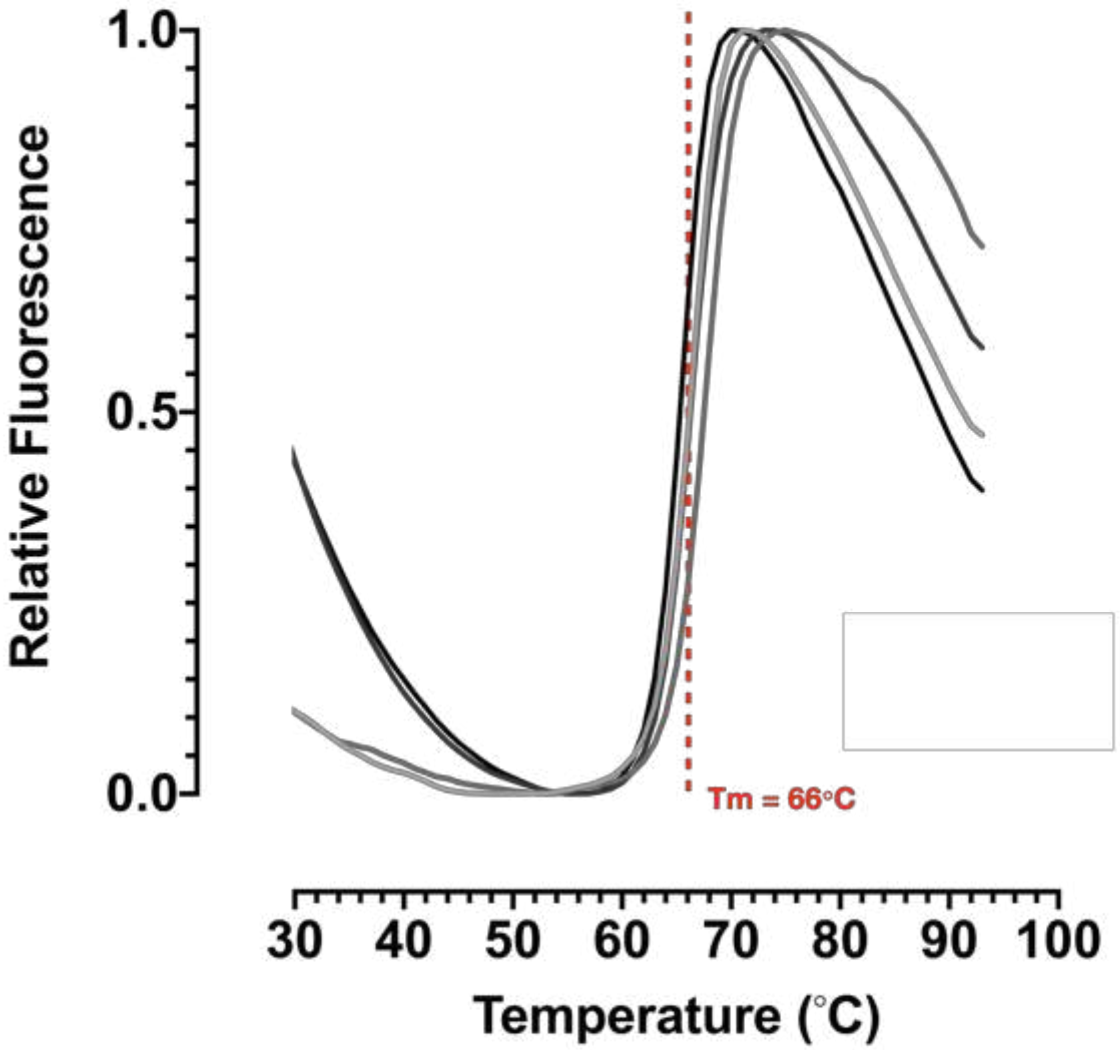

Fig. S8 – Melting curve using differential scanning fluorescence for BtCDPS
Melting curve for BtCDPS in storage buffer, shows a high melting temperature of 66°C. Curves show independent replicates, n=4.

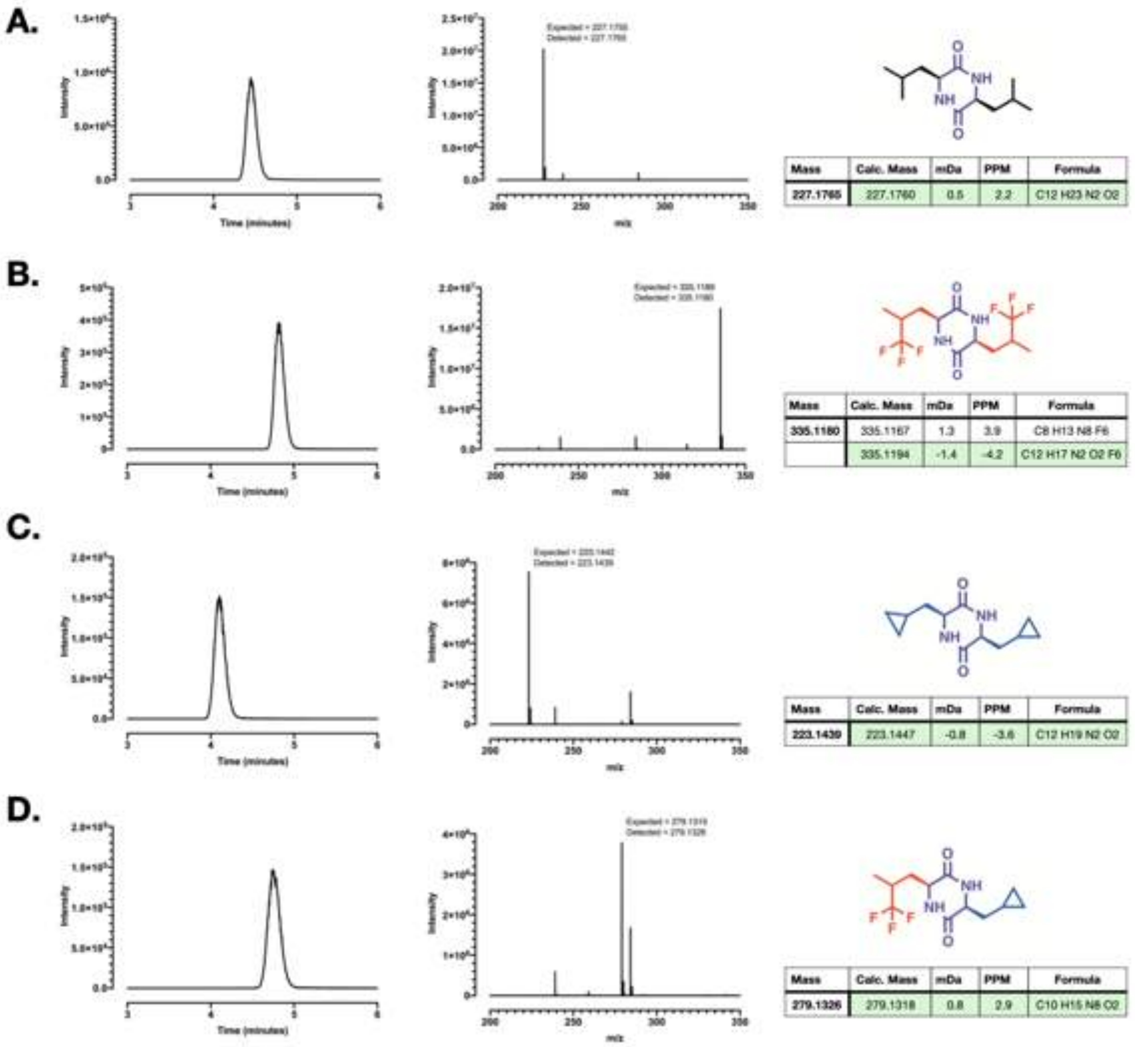

Mass	Calc. Mass	mDa	PPM	Formula
227.1765	227.1760	0.5	2.2	C12 H23 N2 O2

Mass	Calc. Mass	mDa	PPM	Formula
335.1180	335.1167	1.3	3.9	C8 H13 N8 F6
	335.1194	-1.4	-4.2	C12 H17 N2 O2 F6

Mass	Calc. Mass	mDa	PPM	Formula
223.1439	223.1447	-0.8	-3.6	C12 H19 N2 O2

Mass	Calc. Mass	mDa	PPM	Formula
279.1326	279.1318	0.8	2.9	C10 H15 N6 O2

Fig. S9 – HRMS spectra for unnatural aa containing cyclic dipeptides.
Examples of Mass spec results for BtCDPS assays. (A) Detection of cyclic leucine (cLL), (B) detection of cyclic tri-fluoro-leucine, (C) detection of Cyclic cyclo-penta-alanine, (D) detection of cyclic tri-fluoro-leucine cyclo-penta-ala. Panels from left to right represent the TIC for extracted expected mass for each product (calculated from ChemDraw) with a ±0.005Da window, the m/z intensity contribution over this region and calculated elemental composition for the detected mass. All masses reported are mass +1H.

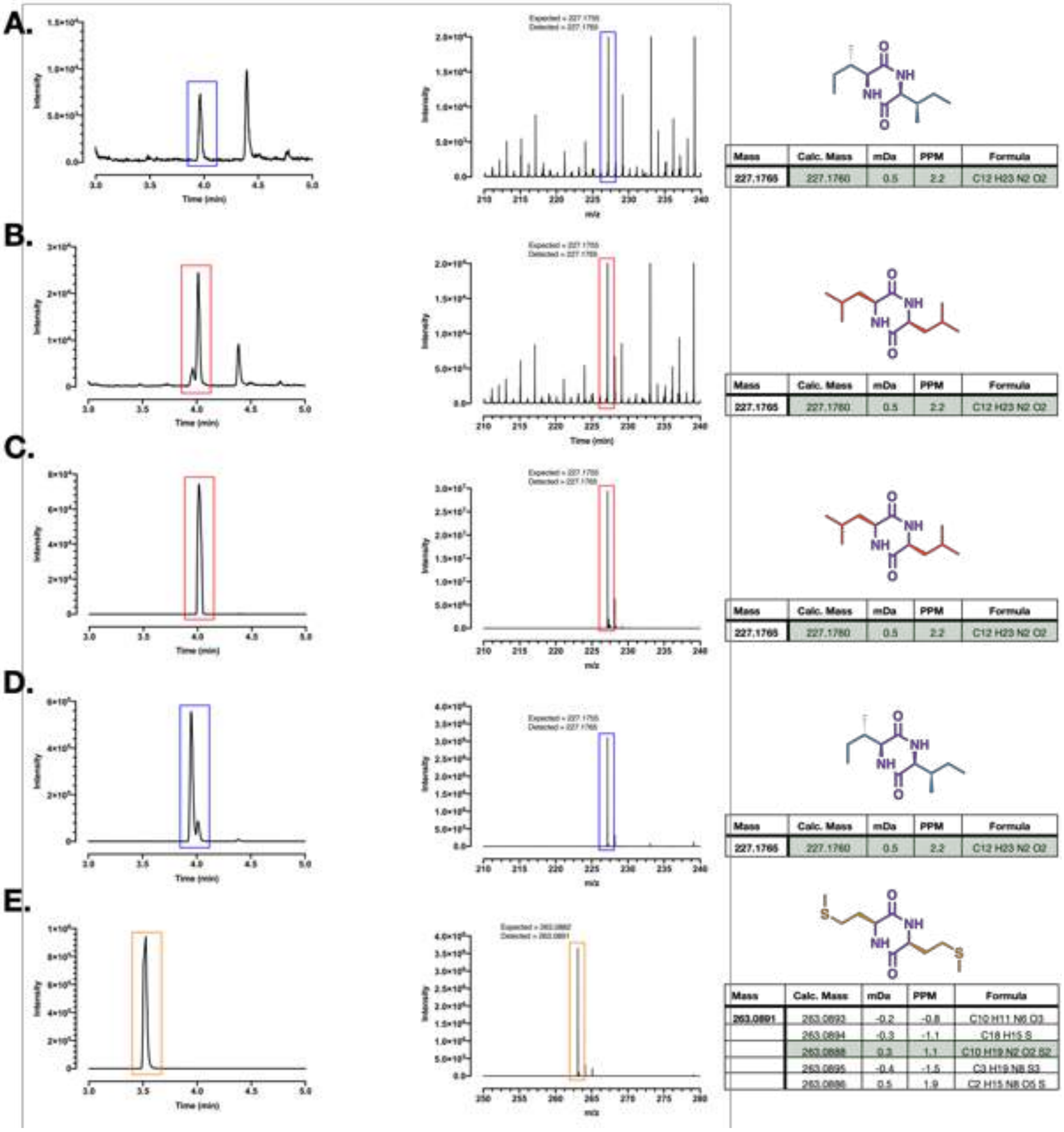

Mass	Calc. Mass	mDa	PPM	Formula
227.1765	227.1760	0.5	2.2	C12 H23 N2 O2

Mass	Calc. Mass	mDa	PPM	Formula
227.1765	227.1760	0.5	2.2	C12 H23 N2 O2

Mass	Calc. Mass	mDa	PPM	Formula
227.1765	227.1760	0.5	2.2	C12 H23 N2 O2

Mass	Calc. Mass	mDa	PPM	Formula
227.1765	227.1760	0.5	2.2	C12 H23 N2 O2

Mass	Calc. Mass	mDa	PPM	Formula
263.0891	263.0893	-0.2	-0.8	C10 H11 N6 O3
	263.0894	-0.3	-1.1	C18 H15 S
	263.0888	0.3	1.1	C10 H19 N2 O2 S2
	263.0895	-0.4	-1.5	C3 H19 N8 S3
	263.0886	0.5	1.9	C2 H15 N8 O5 S

Fig. S10 – tRNA misaminoacylation experiment

Mutant LeuRS-D345A and IleRS-D342A, lacking editing activity were used to misaminoacylate *$tRNA^{Leu}$ and $tRNA^{Ile}$*, respectively with the amino acids leucine, isoleucine, methionine and valine. (A) detection of cII from Ile-$tRNA^{Ile}$, (B) detection of cLL from Leu-$tRNA^{Ile}$, (C) detection of cLL from Leu-$tRNA^{Leu}$, (D) detection of cII from Ile-$tRNA^{Leu}$, (E) detection of cMM from Met-$tRNA^{Leu}$. cMM was not detec ted from Met-$tRNA^{Ile}$. cVV was not detected from either Val-$tRNA^{Ile}$ or Val-$tRNA^{Leu}$. Panels from left to right represent the TIC for extracted expected mass for each product (calculated from ChemDraw) with a ±0.005Da window, the m/z intensity contribution over this region and calculated elemental composition for the detected mass. All masses reported are mass +1H.

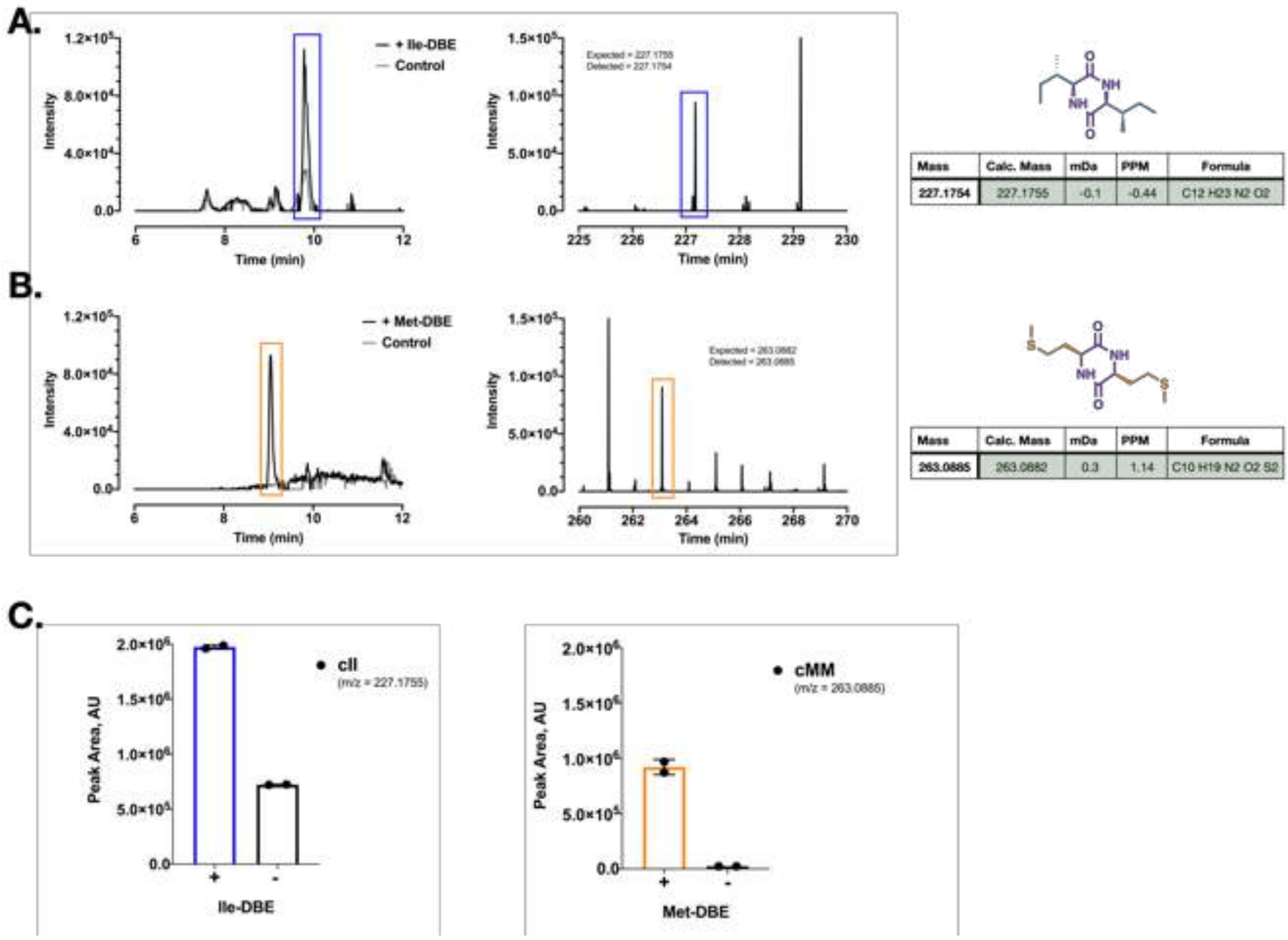

Fig. S11 – Alternative aa-DBE substrates

The ability of BtCDPS to produce cyclic di-peptides from alternative aa-DBE compounds was tested, and the experiment revealed BtCDPS can accept DBE substrates loaded with alternative amino acids (not just leucine). (A) cII was produced by BtCDPS in the presence of Ile-DBE. (B) cMM was produced by BtCDPS in the presence of Met-DBE. (C) The peak area of cyclic di-peptide detected (cII – left, cMM – right) compared to a control lacking substrate. Panels from left to right (for A and B) represent the TIC for extracted expected mass for each product (calculated from ChemDraw) with a ±0.005Da window, the m/z intensity contribution over this region and calculated elemental composition for the detected mass. All masses reported are mass +1H. Experiments performed in duplicate.

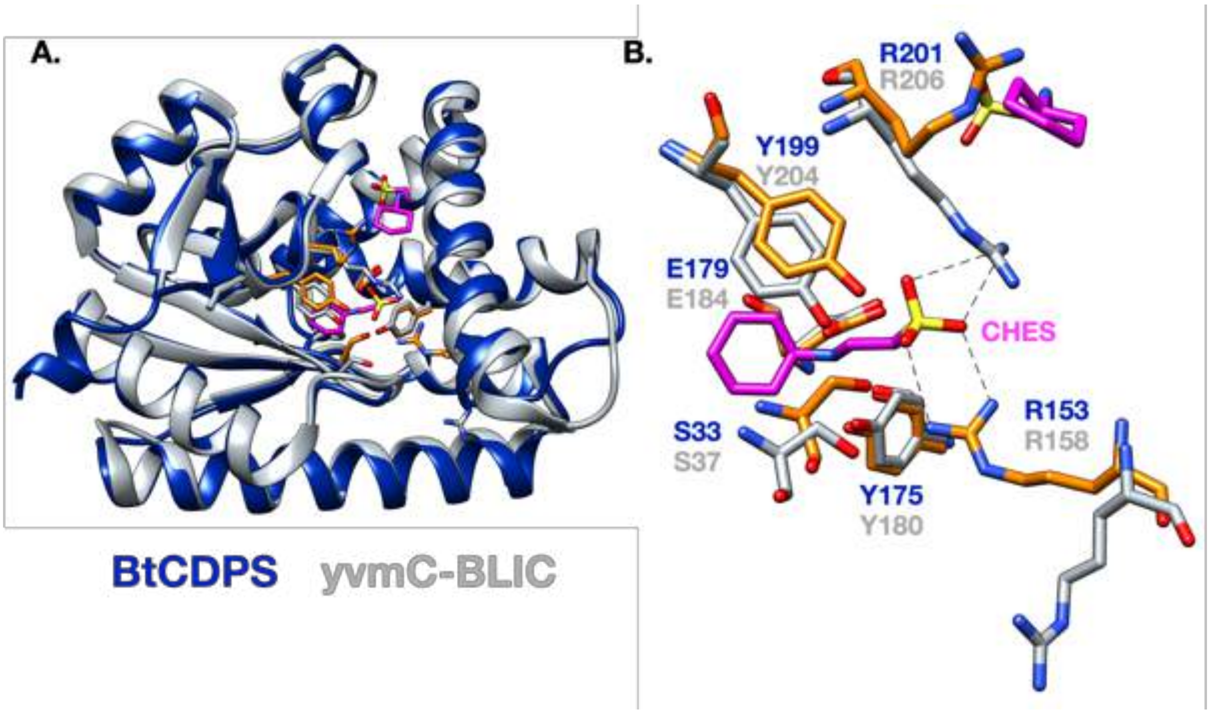

Fig. S12 - Possible role of Arg153

BtCDPS and yvmC-BLIC are close homologues, RMSD 0.88 Å, over 212 residues, sequence ID = 52%. Superimposition with PDB: 3OQI shows position of CHES molecules (ligand mimics) in relation to the active site pocket of BtCDPS. R153 (BtCDPS) and R158 (yvmC-BLIC) occupy the same relative location in the two structures (towards the end of helix-7). However, the sidechain conformation is drastically different between the two structures. This is also notable for R201 (BtCDPS) and R206 (yvmC-BLIC), which H-bonds to the CHES molecule. These arginine residues may therefore be able to sample different conformations in order to interact with the incoming substrate.

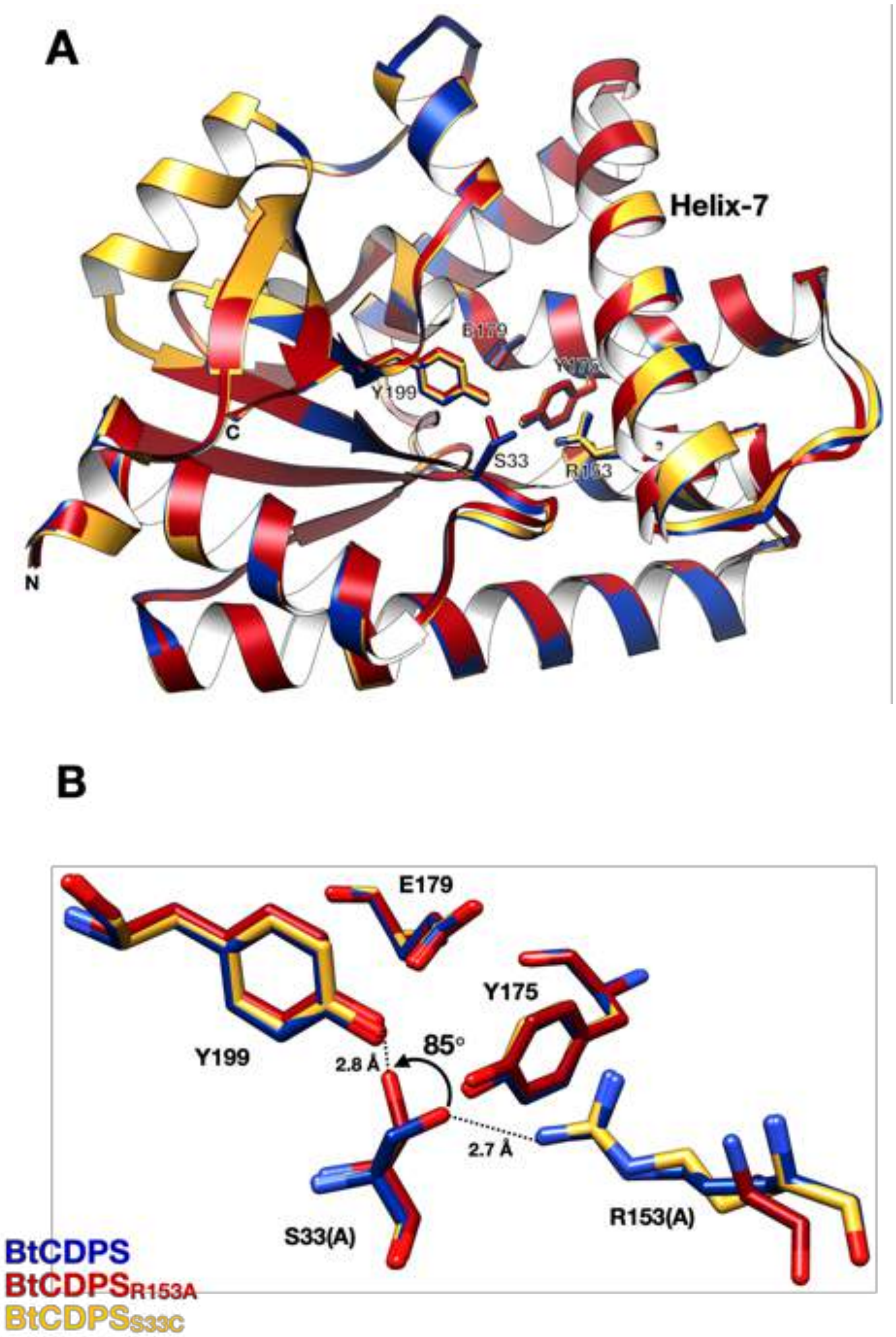

Fig. S13 - BtCDPS mutant structures

(A) The structure of BtCDPS (blue) overlaid with the structure of BtCDPS$_{S33A}$ (yellow) and BtCDPS$_{R153A}$ (red). Little differences exist between all three structures (RMSD = 0.183 and 0.414 between Wt and S33A & Wt and R153A, respectively); the largest difference is the perturbation of helix-7 in the structure of BtCDPS$_{R153A}$, brought about by the absence of R153. (B) The local affect of the R153A mutation results in the obvious loss of the H-bond to active site S33. The sidechain of S33 adpots an alternative conformation in the structure of BtCDPS$_{R153A}$, pivoting ~85° to form a H-bond with active site Y199 instead. The loss of activity in BtCDPS$_{R153A}$ is due, in two parts; incorrect positioning of the active site serine residue and loss of substrate-enzyme interactions.

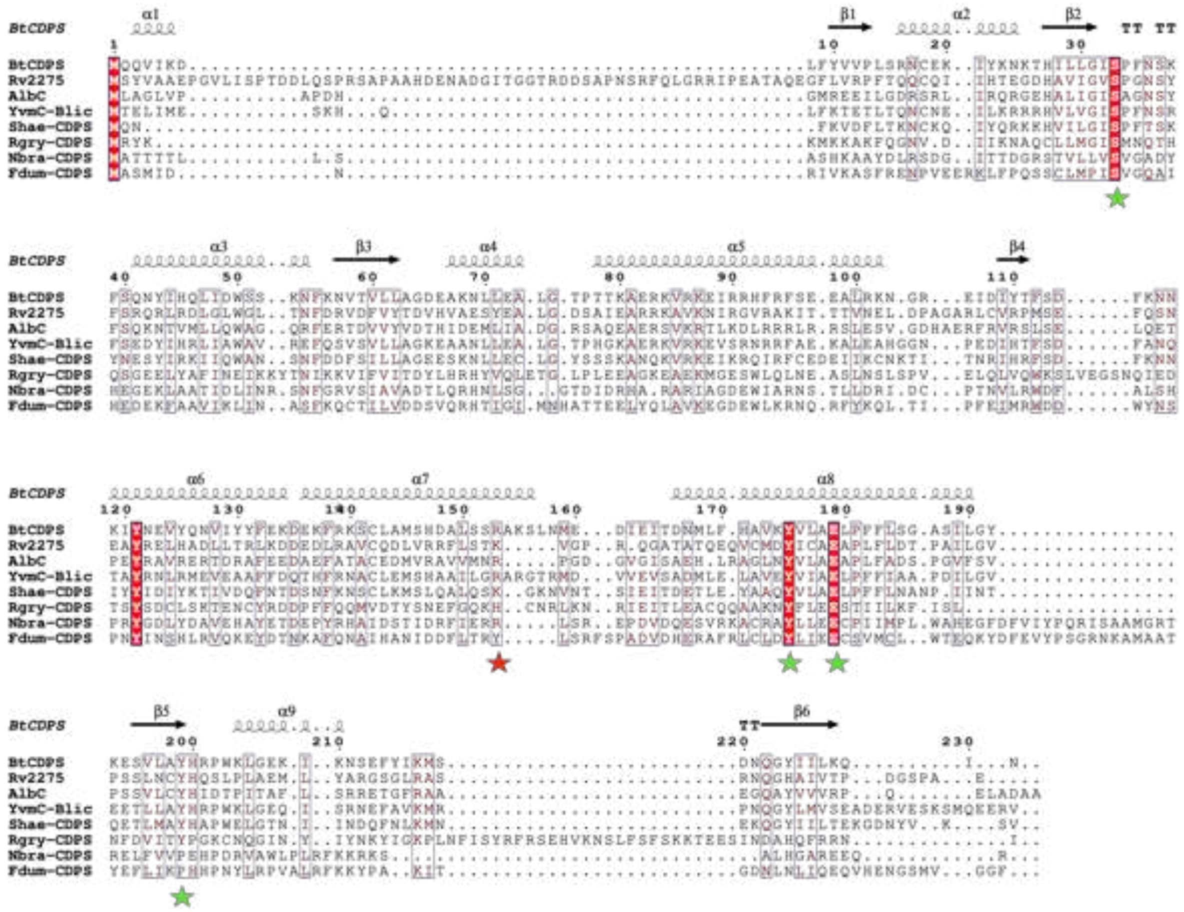

Fig. S14 - Sequence alignment of characterised CDPS

Sequence alignment of BtCDPS and other structurally characterised CDPS enzymes. Strictly conserved residues of the CDPS family are highlighted in red. Green stars highlight the position of the catalytic residues and the red star highlights the position of Arg153. The secondary structure of BtCDPS is shown above the alignment. The alignment was created using TCOFFEE expresso structural alignment tool and drawn using espript. BtCDPS = *Bacillus thermoamylovorans* (6ZTU), Rv2275 = *Mycobacterium tuberculosis* (2X9Q), AlbC = Streptomyces noursei (4Q24), YvmC-Blic = *Bacillus licheniformis* (3OQI), Shae-CDPS = *Staphylococcus haemolyticus* (6EZ3), Rgry-CDPS = *Rickettsiella grylli* (5MLP), Nbra-CDPS = *Nocardia brasiliensis* (5MLQ), Fdum-CDPS = *Fluoribacter dumoffii* (5OCD)

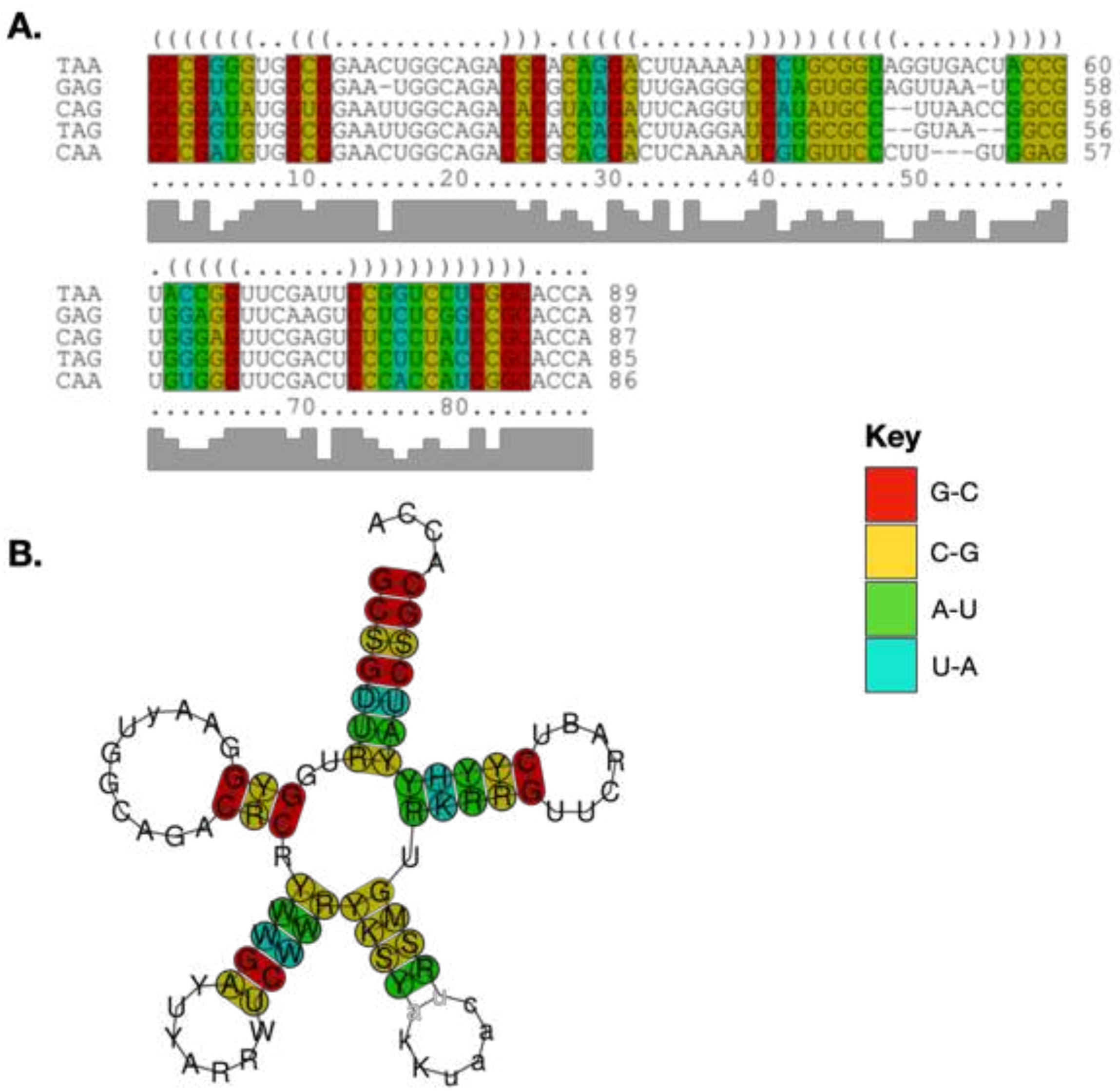

Fig. S15 - Sequence alignment of tRNA^{Leu} from Bacillus thermoamylovorans

Alignment of tRNA^{Leu} isoacceptors RNA sequences was created using Freiburg RNA Tools LocARNA. (A) The RNA primary sequence alignment, brackets above show bases involved in predicted secondary structure. Grey bar chart underneath alignment illustrates the conservation of bases. Colours bases conform to the key and represent the type of compatible bonds formed. (B) The consensus RNA fold tRNA^{Leu} isoacceptors alignment, with colours corresponding to the compatible bonds key.

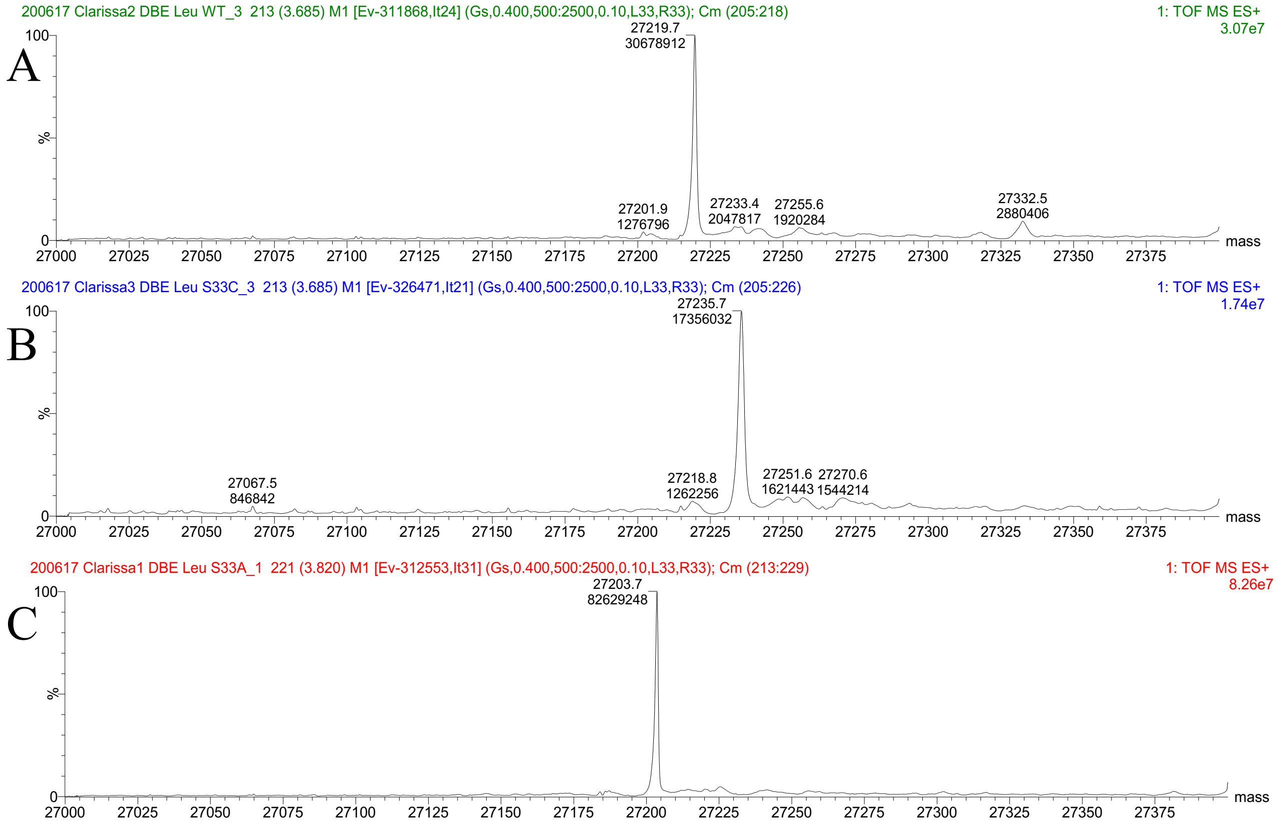

Fig. S16 – Intact mass spec of S33A and S33C

Intact mass spectroscopy results for BtCDPS (A), $BtCDPS_{S33C}$ (B) and $BtCDPS_{S33A}$ (C) after incubation with 2.5mM Leu-DBE. Only BtCDPS has a peak at m/z 27332.5, corresponding to an approximate mass increase of +115 (Leucyl intermediate).

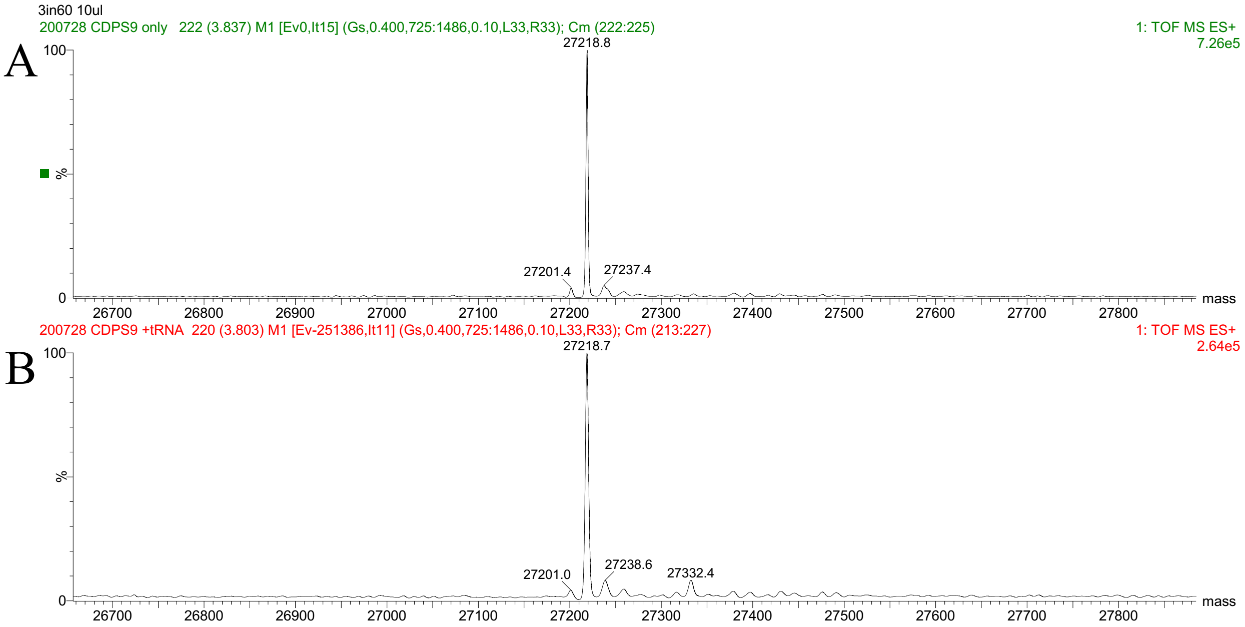

Fig. S17 – Intact mass spec of BtCDPS - tRNA assay

Intact mass spectroscopy results for BtCDPS (A), BtCDPS – $tRNA^{Leu}$ (B) and BtCDPS + $tRNA^{Leu}$. A control cyclodipeptide synthesis assay was carried out in the presence of $tRNA^{Leu}$, leucine and LeuRS to determine whether a substantial amount of a Leucyl-enzyme intermediate species is formed when Leu-$tRNA^{Leu}$ is used as the substrate. This control confirms a large proportion of BtCDPS becomes a trapped leucyl-enzyme intermediate when Leu-DBE substrate is used as a substrate.

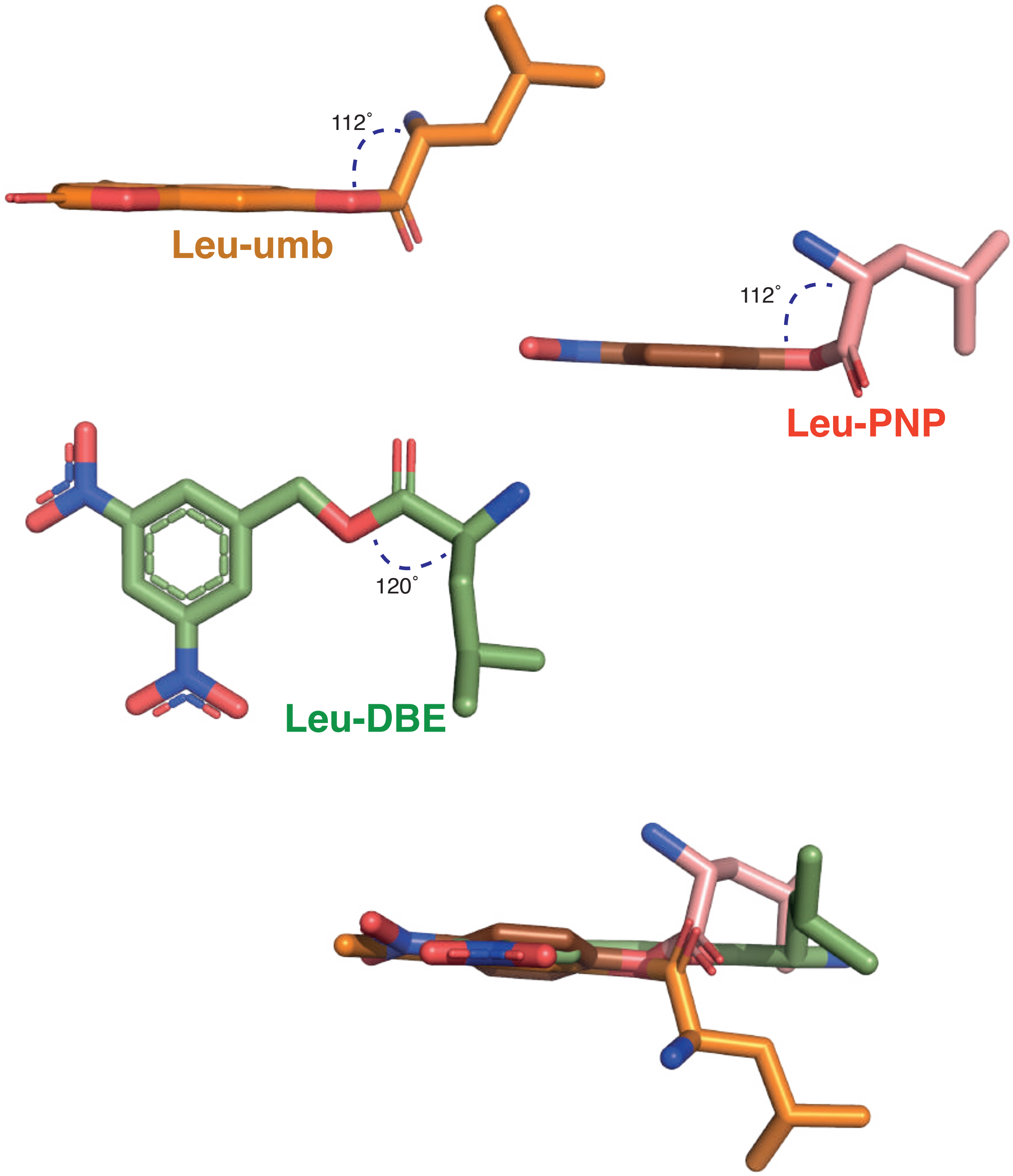

Fig. S18 – Comparison between minimal substrates
Comparison between energy minimized structures of Leu-Umb, Leu-PNP ad Leu-DBE, generated using ChemDraw 3D. Angle between oxygen, carbonyl carbon and carbon linked to amine is shown for comparison. The dihedral angle between the C-C bond of leucine was calculated to produce a structure with minimised steric hindrance. In addition, MM2 optimisation was performed on the entire molecule.

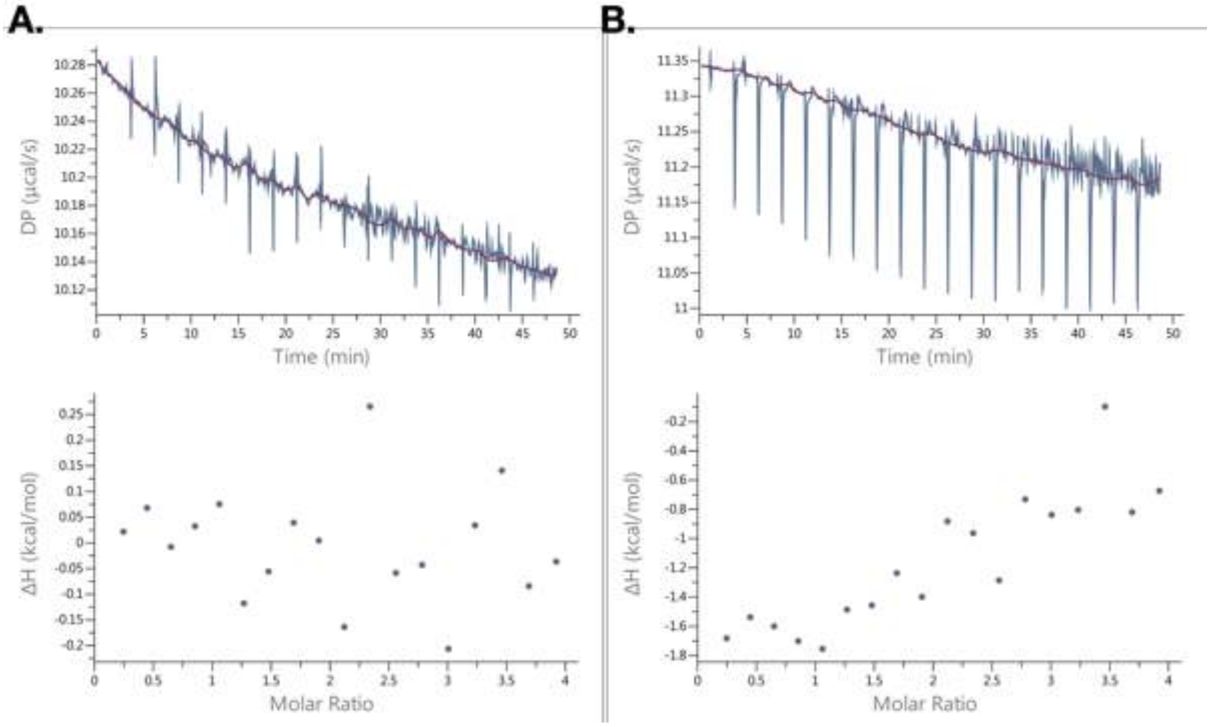

Fig. S19 – ITC data
ITC was performed to assess binding between BtCDPS and Leu-PANS, although no binding was detected. (A) BtCDPS at 50 µM in the cell titrated with Leu-PANS in the syringe at 2mM over 19 injections. (B) Reverse titration, Leu-PANS at 25 µM in the cell titrated with BtCDPS in the syringe at 500 µM over 19 injections.

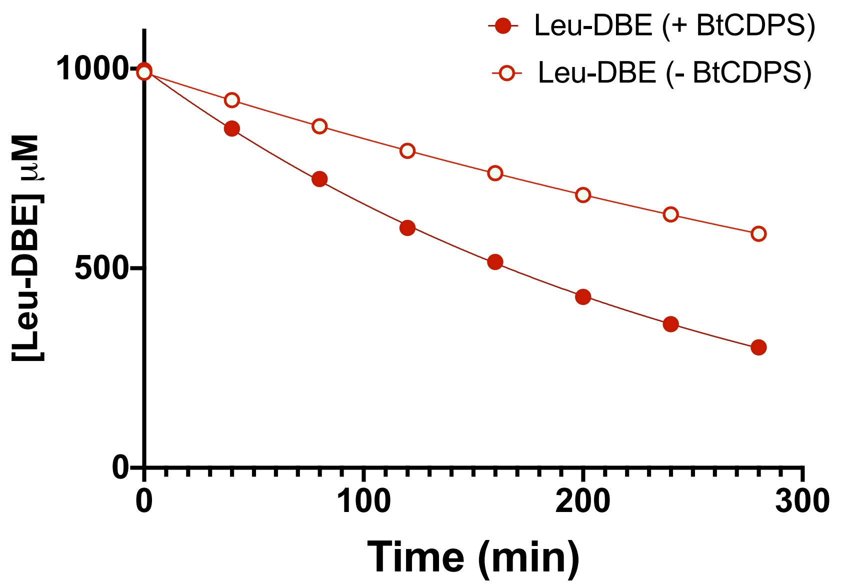

Fig. S20 – Leu-DBE hydrolysis with and without BtCDPS.

Leu-DBE concentration change in the presence and absence of BtCDPS was monitored by HPLC and quantified using peak areas with absorbance 220 nm. Retention times were determined using standards. Line is a fit to an exponential decay equation with format Y=(Y0 - Plateau)*exp(-K*X) + Plateau, where Y0 is the initial Y value, plateau is the asymptotic y value when x approaches infinity, K is the decay rate constant, and the half life is the time in which Y0 – Plateau = ½. Values obtained after fitting are:

	**Yo**	**Plateau**	**K**	**Half life**
**Leu-DBE (- BtCDPS)**	990.21 ± 2.47	-122.06 ± 107.34	0.00161 ± 0.00019	430.87
**Leu-DBE (+ BtCDPS)**	998.50 ± 5.26	-67.63 ± 40.94	0.00380 ± 0.00024	182.29

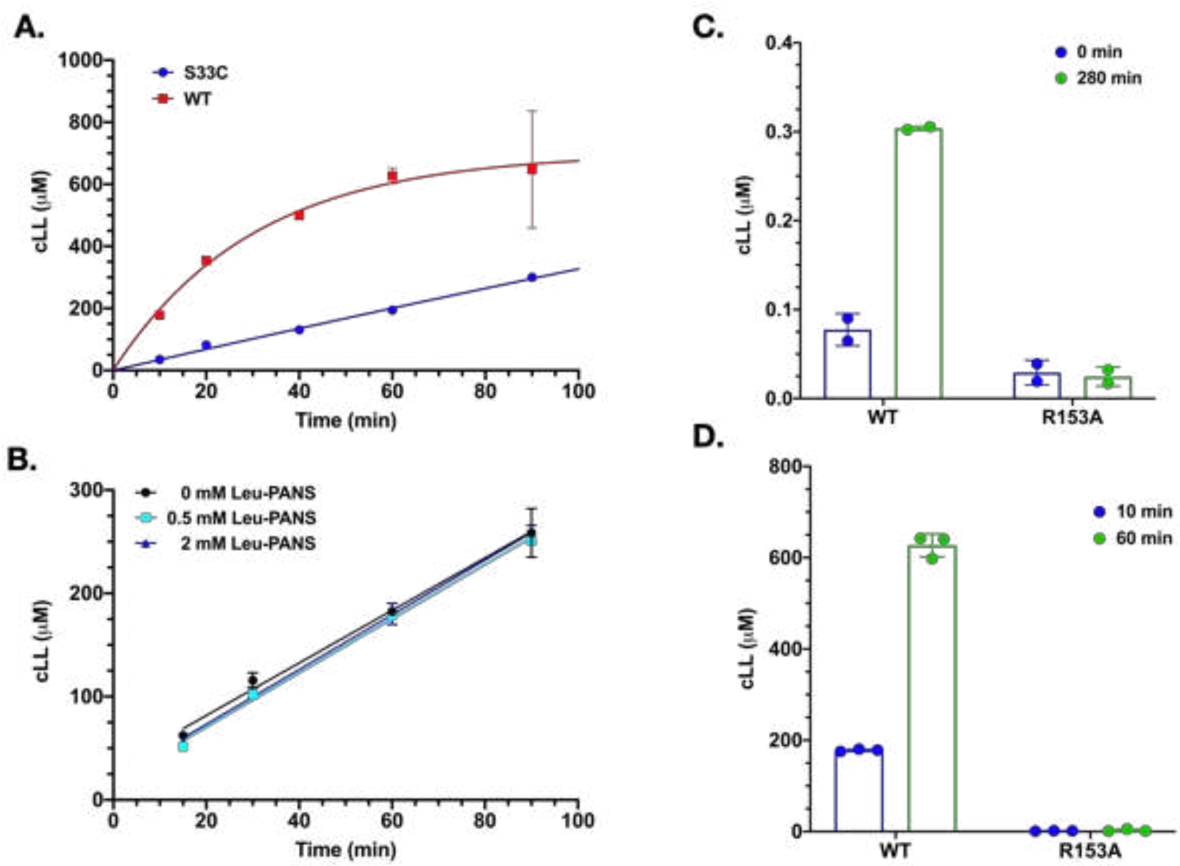

Fig. S21 – tRNA assay timecouse, Leu-PANS inhibition, R153A activity

(A) A timecourse assay monitoring the formation of cLL by LC-MS was performed for WT BtCDPS and S33C and R153A mutants. No activity was detected for the R153A mutants. Data is a fit to an exponential decay equation with format Y=(Y0 - Plateau)*exp(-K*X) + Plateau, where Y0 is the initial Y value, plateau is the asymptotic y value when x approaches infinity, K is the decay rate constant, and the half life is the time in which Y0 – Plateau = ½. Values obtained after fitting are below (B)An tRNA timecourse assay was performed for BtCDPS in the presence of different concentrations of Leu-PANS. The presence of Leu-PANS did not affect the rate of cLL formation suggesting it is not an inhitor of BtCDPS. Data fit to simple linear regression, values shown below. (C) Comparison of cLL formation by Wt and R153A BtCDPS at two separate timepoints in the Leu-DBE assay. (D) Comparison of cLL formation by Wt and R153A BtCDPS at two separate timepoints in the tRNA assay. R153A is inactive.

**Enzyme variant**	**Yo**	**Plateau**	**K**	**Half life**
**WT**	0	700.93 ± 50.67	0.0331 ± 0.0057	20.96
**S33C**	0	3127.68 ± 3878.97	0.00110 ± 0.0014	627.7942

**[Leu-PANS]**	**Slope**
**0 mM**	2.54 ± 0.15
**0.5 mM**	2.62 ± 0.07
**2 mM**	2.65 ± 0.08

Table S3. Crystallographic data

	BtCDPS	$BtCDPS_{S33A}$	$BtCDPS_{R153A}$
**Accession Code**	6ZTU	6ZU3	7AZU
**Data Collection**			
Spacegroup	C 2	C 2 2 2_1	P 4_1 2_1 2
**Unit Cell Dimensions**			
Cell Dimensions a, b, c (Å)	80.0, 80.0, 92.1	79.8, 79.8, 91.9	79.8, 79.8, 92.8
α, β, γ (°)	90, 90, 90	90, 90, 90	90, 90, 90
Resolution (Å)	56.5-1.69	60.3-1.78	48.2-1.80
Total Reflections	857699 (41424)	633528 (31730)	749431 (32495)
Unique Reflections	34140 (2485)	24909 (1789)	28430 (1360)
Rmerge(I)	0.096 (2.811)	0.059 (2.533)	0.059 (2.139)
Rpim	0.019 (0.699)	0.012 (0.612)	0.012 (0.440)
I/sigI	19.4 (1.1)	28.3 (1.1)	26.4 (1.4)
CC(1/2)	1.00 (0.488)	1.00 (0.611)	1.00 (0.833)
Completeness (%)	100 (100)	100 (100)	100 (97.8)
Multiplicity	25.1 (16.7)	25.4 (17.7)	26.4 (23.9)
**Refinement**			
R-work (%)	18.96	18.30	20.41
R-free (%)	21.19	21.96	22.59
Average B Factor (Å^2)	33.9025	45.9466	47.4758
No. of Atoms	2087	2012	2020
Protein	1918	1920	1904
Ligand / Ion	8	0	8
Solvent	161	92	107
Molecules per ASU	1	1	1
RMS (bonds) (Å)	0.005	0.006	0.006
RMS (angles) (°)	0.759	0.757	0.798
**Ramachandran Statistics**			
Favoured (%)	98.25	97.37	97.81
Allowed (%)	1.75	2.63	2.19
Outliers (%)	0	0	0

*Numbers in parentheses refer to the outermost shell

Supporting References

1. S. R. Starck, X. Qi, B. N. Olsen and R. W. Roberts, *Journal of the American Chemical Society*, 2003, **125**, 8090-8091.
2. B. R. Baker, J. P. Joseph and J. H. Williams, *Journal of the American Chemical Society*, 1955, **77**, 1-7.
3. J. R. Peacock, R. R. Walvoord, A. Y. Chang, M. C. Kozlowski, H. Gamper and Y. M. Hou, *Rna-a Publication of the Rna Society*, 2014, **20**, 758-764.
